# Magnetic Resonance Imaging and Iron-oxide Nanoparticles in the era of Personalized Medicine

**DOI:** 10.7150/ntno.86467

**Published:** 2023-08-21

**Authors:** Mahbuba Rahman

**Affiliations:** Department of Biology, McMaster University, Hamilton, Ontario, Canada.

**Keywords:** Magnetic resonance imaging, nanotechnology, biomarkers, personalized medicine, iron-oxide nanoparticles.

## Abstract

Medical imaging is an important factor for diagnosis. It can be used to diagnose patients, differentiate disease stages, and monitor treatment regimens. Although different imaging technologies are available, MRI is sensitive over other imaging modalities as it is capable of deep tissue penetration allowing to image the anatomical, structural, and molecular level of diseased organs. Thus, it can be used as screening tool for disease staging. One of the important components of imaging is contrast agents which are used to increase the sensitivity of MRI technology. While different types of contrast agents are available, iron-oxide based nanoparticles (IONPS) are widely used as these are easy to formulate, functionalize, biocompatible and cost effective. In addition to its use as contrast agents, these have been used as drug carriers for the treatment of different types of diseases ranging from cancer, cardiovascular diseases, neurological disorders, autoimmune diseases, and infectious diseases. For the last two decades, there has been advancement in nanotheranostics, where IONPs are formulated to carry drug and be used as contrast agents in one system so that these can be used for image-guided therapy and monitor real-life treatment response in diseased tissue. This technology can be used to stratify patients into responders and non-responders and reduce adverse drug toxicity and lead to a tailored treatment. However, success of nanotheranostics depends on several factor, including identification of disease associated biomarkers that can be targeted on IONPs during formulation. While many challenges exist for the clinical translation of nanotheranostics, it still has the potential to be implemented in personalized treatment strategy. In this review article, we discussed the use of MRI technology and IONPs in relation to their application in disease diagnosis and nanotheranostics application in personalized medicine.

## Introduction

Detecting the disease before clinical symptoms appear can save lives. When a patient presents with the first signs and symptoms of disease, in most cases the diagnostic pathway is through medical imaging combined with biological fluid analysis in the laboratory 1-3. Medical imaging has long been used to diagnose diseases in noninvasive manner. In clinics, it is used as disease screening tool. Screening can at least lead to the assumptions to segment patients into low-, intermediate-risk, and low-risk subgroups, and high-risk subgroups 1,2. Imaging additionally plays an important role in monitoring, follow-up planning, and targeting of non-invasive treatment regimens in patient-tailored manner. This is expected to bring changes in clinical practice, research and for future professionals of the imaging community 4,5.

There are many types of image processing. These include Positron Emission Tomography (PET), Computed Tomography (CT), Ultrasound, Single Photon Emission Computed Tomography (SPECT), Magnetic Resonance Spectroscopy (MRS) and magnetic resonance imaging (MRI) 6,7. Among the various imaging modalities, MRI offers deep imaging of soft tissues, allowing it to detect changes in molecular and structural abnormalities 8. In the 1970s, Freeman *et al*. were the ones who initially proposed the idea of using magnetism in medicine 9. Paul Lauterbur published the first MR image in 1973 and the first research conducted on humans were published in 1977 10. In the early 1980s, magnetic resonance imaging (MRI) was introduced into clinics 16, 17. The use of MRI in clinical oncology has grown significantly over the last few decades. In addition, MRI has been used to detect host-pathogen interaction in animal model. The purposes of some of these investigations were to understand the mechanisms of diseases for drug development and to understand the mechanisms of drug resistance. MRI is also used to investigate medical conditions that may develop post-infection and may cause severe health condition in patients 11. In this way, MRI has the potential as diagnostic tool for diseases where multiple organ systems are affected 11. However, due to less sensitivity, contrast agents are used for imaging. Contrast enhanced MRI offers the ability to identify and detect disease-related biomarkers in correlation to the precise anatomical location. MRI can reveal the molecular events behind the onset and course of disease when used in conjunction with the appropriately targeted MRI contrast agents 10. At present, nanoparticles are used with contrast agents and there has been advancement on the use of nanotechnology to synthesize nanoprobes that can be used both for drug delivery or diagnosis 12,13. The size of the nanoparticles range from ~10 to 100 nm, and no larger than 1000 nm 14. Nanotechnology-based drug delivery systems ensures safe drug delivery in the body, as their size allows for drug delivery by intravenous injection or other methods. In addition, the nano-size also reduces the pathogen's response at the injection site 5. Their size has the great advantage to supply a large amount of material to the targeted site 15. Several nanoparticle-based treatment and diagnosis are available for cancer, cardiovascular diseases, autoimmune diseases, neurodegenerative diseases, pulmonary diseases and also for infectious diseases 12. While these shows promises on the use of nanotechnology in the area of non-communicable and for communicable diseases, application of nanotechnology is still at its infancy in the use of targeted diagnostics and combination of therapy and imaging, known as nanotheranostics 16,17.

Nanoparticles are of different types, organic and inorganic. Among the inorganic nanoparticles, iron-oxide based superparamagnetic iron oxide nanoparticles (SPIONs) have demonstrated significant promise as contrast agent for MRI. Numerous biomedical applications are made possible by the inherent characteristics of SPIONs, including their built-in magnetism, high margin of safety, and accessibility to fabrication and surface engineering techniques. SPIONs can achieve the highest drug targeting efficiency among other nano-carriers because for a localized application of external magnetic field to the target organ which facilitates the accumulation of magnetic nanoparticles' at the drug's site of action 16,17. This combination of MRI and nanotechnology, can play important role in real-time monitoring of therapeutic outcomes and thereby mitigate multi-drug resistance in case of communicable diseases, antimicrobial drug resistance in case of infectious diseases and reduce drug toxicity. However, research shows that patient survival, treatment response and drug toxicity vary widely among patients and these are related to the environmental factors, genetic profile and signaling molecules of the immune system that consists of proteins, metabolites, and co-factors of the patient. Therefore, current treatment strategy, which is based on “one-size fits all,” requires a patient tailored treatment strategy, namely “personalized medicine” (PM). Introduced by Leroy Hood, personalized medicine offers deeper understanding of pathological conditions more precisely using blood as a non-invasive biosample. In addition to blood, other biological fluids are also used to segregate disease stage and treatment response to predictive, prognostic, personalized and participatory treatment strategy 3. Success of this treatment strategy requires identification of biomarkers that are robust enough to correlate diseased stage or treatment response in patient tailored manner and thereby reduce adverse drug toxicity. In clinical setting, a biomarker should be quantifiable from biospecimens such as blood, sweat, urine, tissues, or cerebral spinal fluid (CSF). In the specific context of diagnostic imaging biomarkers, the expression of biosignal that can be detected and evaluated utilizing electromagnetic, photonic, or acoustic signal emitted by the patient are considered as imaging biomarkers, in contrast to the biomarkers that are detected from biospecimens mentioned earlier. Imaging biomarkers, being non-invasive, could replace the requirement for assessment of biomarkers that involves invasive assessment of biospecimens, for example, tissue biopsy or CSF 18. For MRI based imaging technology, the biomarkers also need to fulfill some characteristics such as they should be sensitive, specific and biologically relevant, quantifiable and reproducible and cost-effective 19. Due to the favorable characteristics of iron-oxide based SPIONs, mentioned above and also of its cost-effectiveness, these can be used in disease diagnosis and also as nanotheranostics 19,20. Considering the importance of this technology, in this review article, we discussed the MRI technology and nanoparticles that are used for drug delivery and imaging, followed by their challenges in personalized medicine.

## Magnetic Resonance Imaging

Over the past 40 years, various improvements have been made in magnetic resonance (MR) techniques, which enabled this technology to be used as non-invasive primary disease diagnosis modality in clinics 21,22.

**Figure 1:**
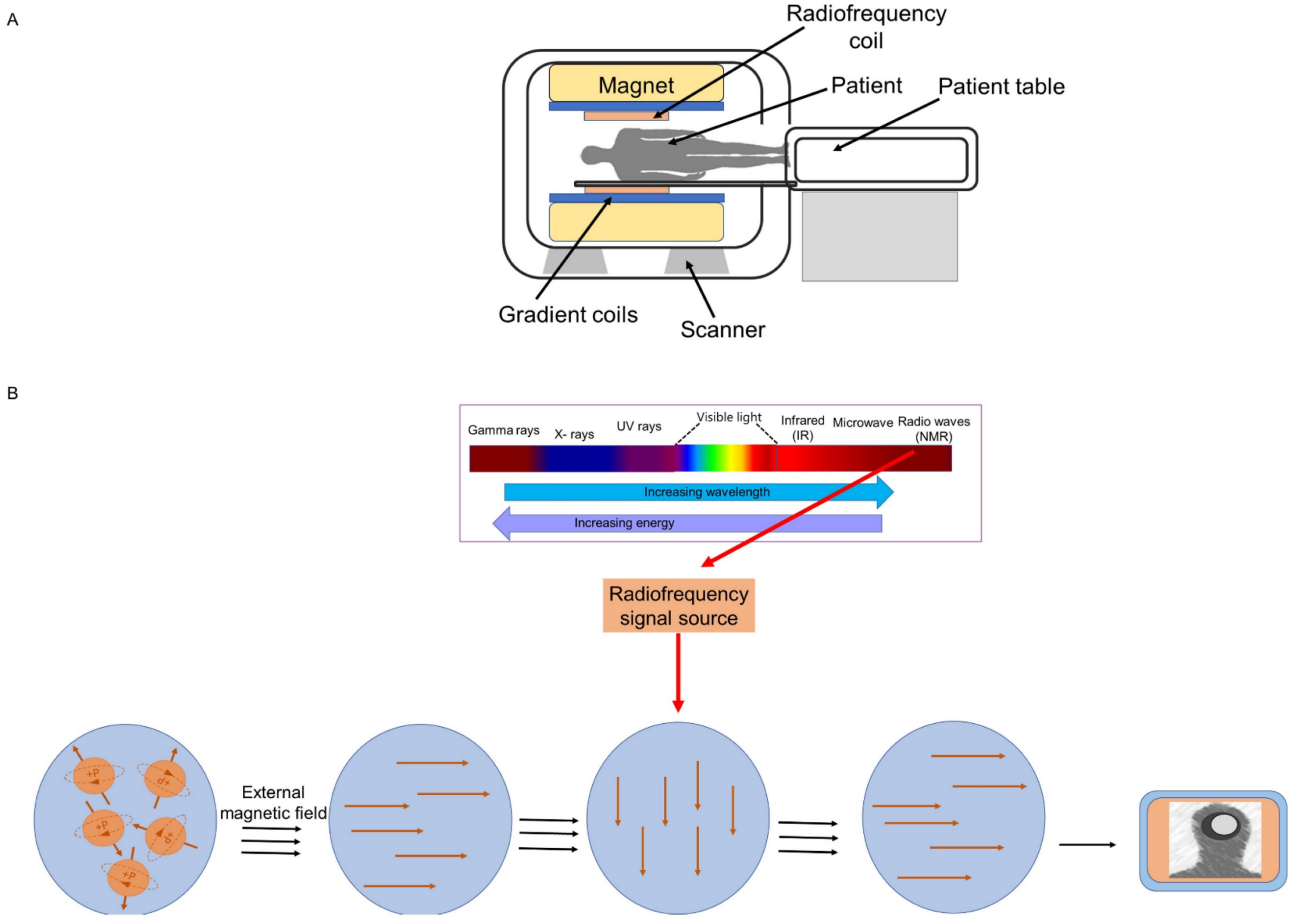
** MRI scanner** (A) and A graphical representation of the acquisition of the MRI signal (B).

MRI employs nuclear magnetic resonance (NMR) to obtain high-resolution images of the internal structure (tissues) of the body. Every tissue contains 70 to 90 percent water that contains hydrogen proton molecule and in the absence of an external magnetic field, the protons are randomly oriented. Most diseases manifest themselves by an increase in water content. So, MRI is a sensitive test for the detection of disease. To obtain image, a patient or biomedical sample is placed inside the MR scanner. An MRI system is composed of main magnet, gradient coils, radiofrequency coils and computer system that control and interface various components (Figure 1A). Each component interacts with the others in a complex relationship 23. The object placed inside the scanner is exposed to an external magnetic field, typically between 0.5 and 3 Tesla in clinical settings and up to 10.5 Tesla in research studies. This aligns the water protons' magnetic poles with the field. The images are created by determining proton relaxation in the presence of an external magnetic field. The orientation of the hydrogen nuclear spins in the presence of a strong magnetic field is parallel or antiparallel to the direction of the magnetic field. The number of excited protons in the high-energy state is augmented by resonant radiofrequency (RF) irradiation, which can transfer energy to the protons. Once the pulsing field stops, the magnetic moments (spin) of protons then return to their original condition, known as relaxation 24.

Transverse magnetization decay (T2-decay) and longitudinal magnetization recovery (T1-recovery) are two distinct relaxation processes. T1 is the amount of time required for the longitudinal magnetization to return to equilibrium and attain 63%, whereas T2 is the amount of time required for the transverse magnetization to exponentially decrease to 37% of its initial magnitude. In other words, the T1 relaxation time is the rate at which excited spinning protons return to equilibrium and realign with the external magnetic field. The T2 relaxation time is the rate at which excited protons lose coherence with each other. Repetition time (TR, or the amount of time between radiofrequency pulse replication), and echo time (TE, time between the radiofrequency pulse and the first signal measurement) are two crucial variables that influence signal intensity and image's contrast. Whereas TE (>10 ms) and TR (>250 ms) are considered short times for T1-weighted images, long TE (>60 ms) and long TR (>2000 ms) are employed to create images for the T2 state (Figure 1B). Various tissues have displayed variable contrasts 25. Tissues with short T1 generate brightness in MRI images, while those with short T2 produce dark images. A T1-weighted scan shows all fat deposits as high intensity pixels in the image. A T2-weighted scan shows all fat and water as high intensity pixels. The two types of images are used together to map organs and diagnose disease. Most tissues do not have enough inherent T1 and T2 relaxation capabilities, therefore affects the quality of the images. Exogenous contrast agent materials are employed to enhance the relaxation time and improve the visibility of the images. Relaxivity (R1 and R2) is a feature that has an impact on the effectiveness of a contrast agent and is defined as the change in the relaxation rate of solvent water protons (R1 = 1/T1 and R2 = 1/T2 in units of s1) when a contrast agent is present. The most employed contrast agents are paramagnetic compounds, particularly gadolinium. Gadolinium is a commonly utilized (T1) contrast agent that, by shortening the T1 relaxation period, boosts signal intensity and results in brighter (T1-weighted) pictures 25. However, the advent of novel contrast materials was spurred due to the low sensitivity, restricted blood flow, and potential toxicity of gadolinium-based contrast agents 24. Manganese (II) complexes, which is another contrast (T1) agent, also possess toxicity for cardio and pulmonary organs. A most recent contrast agent, linked to magnetic contrast agent and nanotechnology is SPION. SPIONs are also known as one type of magnetic nanoparticle (MNP) or iron-oxide nanoparticle (IONPs). These are T2 contrast agents. However, they can be modified to T1 contrast agents 26. Details of iron-oxide based magnetic nanoparticle and their application in imaging, diagnosis and drug delivery are discussed in the following sections.

## Magnetic behavior of Iron-oxide based Nanoparticles

Mentioned earlier, iron-oxide based nanoparticles (IONPs) are used as T2 contrast agents, but their properties can be engineered and used as T1 contrast agent 27. In this section, we will discuss in details about IONPs.

Iron oxide nanoparticles (IONPs) or magnetic nanoparticles (MNPs) are available in several forms. MNPs come in a wide range of forms, including pure metals (Fe, Co, NI, and Mn), metal oxides (Fe_3_O_4_, c-Fe_2_O_3_), metal alloys (FePt, CoPt), and ferrites (MFe_2_O_4_, where M=Co, Cu, Ni, Mn, and Mg). Because of the oxidative characteristics and high toxicity levels of pure metals (and despite their higher Ms=magnetization), they are not suitable for in vivo use. For such reasons, the most widely used magnetic iron oxide nanoparticles are magnetite (Fe_3_O_4_) and maghemite (c-Fe_2_O_3_), both of which have cubic inverse spinel structures and are used in a wide range of health-care sectors 28. These have high chemical/colloidal stability and biocompatibility, and upon surface modification of magnetite and maghemite, the resulting superoxide nanoparticles (SPIONs) can shorten the T1 and T2 proton relaxation times. This properties enable SPIONs to be widely used to enhance the contrast of MR images and apply for magnetic resonance imaging, hyperthermia, targeted delivery of drugs, proteins, antibodies, nucleic acids, biosensing, tissue repair and separation of biomolecules 16,29.

In the following section, we discussed the physicochemical properties that play important role for IONPs to be used in diagnostics and drug delivery.

## Physicochemical properties of Iron-oxide based Nanoparticles for diagnostics and drug delivery

The physicochemical properties of IONPs that are associated with magnetic behavior and needs to be considered for disease diagnosis or drug delivery are shape, size, charge, surface modifications, coating, drug loading, preparation, functionalization and, targeting approach. Nanoparticles to be used for biomedical application needs to be chemically stable, homogenous in size, and well distributed in liquid media. Researchers also aim to optimize the properties of magnetic particles so that their concentration can be increased in blood vessels, reduce early clearance from the body, minimize non-specific cell interactions and thereby minimize side effects, increase the efficiency of the internalization within target cells and decrease total dose of the drug 30,31. Several review articles have explained in detail about the functionalization and preparation methods 16,29. Here we have discussed some of the properties that govern the blood distribution profile and needs to be considered for application in diagnosis and drug delivery.

### Size

One of the most significant attributes of nanoparticles is their particle size, which has a significant impact on both their biomedical applications as well as the magnetic properties of MNPs or IONPs. The size of IONPs varies depending on the production method used and the coating and can range from < 10 nm to over 100 nm. IONPs below 10 nm are known as very small superparamagnetic iron oxide NPs (VSPIONs); those between 10 and 50 nm are known as ultrasmall superparamagnetic iron oxide NPs (USPIONs), and those between 50 and 180 nm are known as superparamagnetic iron oxide nanoparticle (SPIONs) 32.

**Figure 2:**
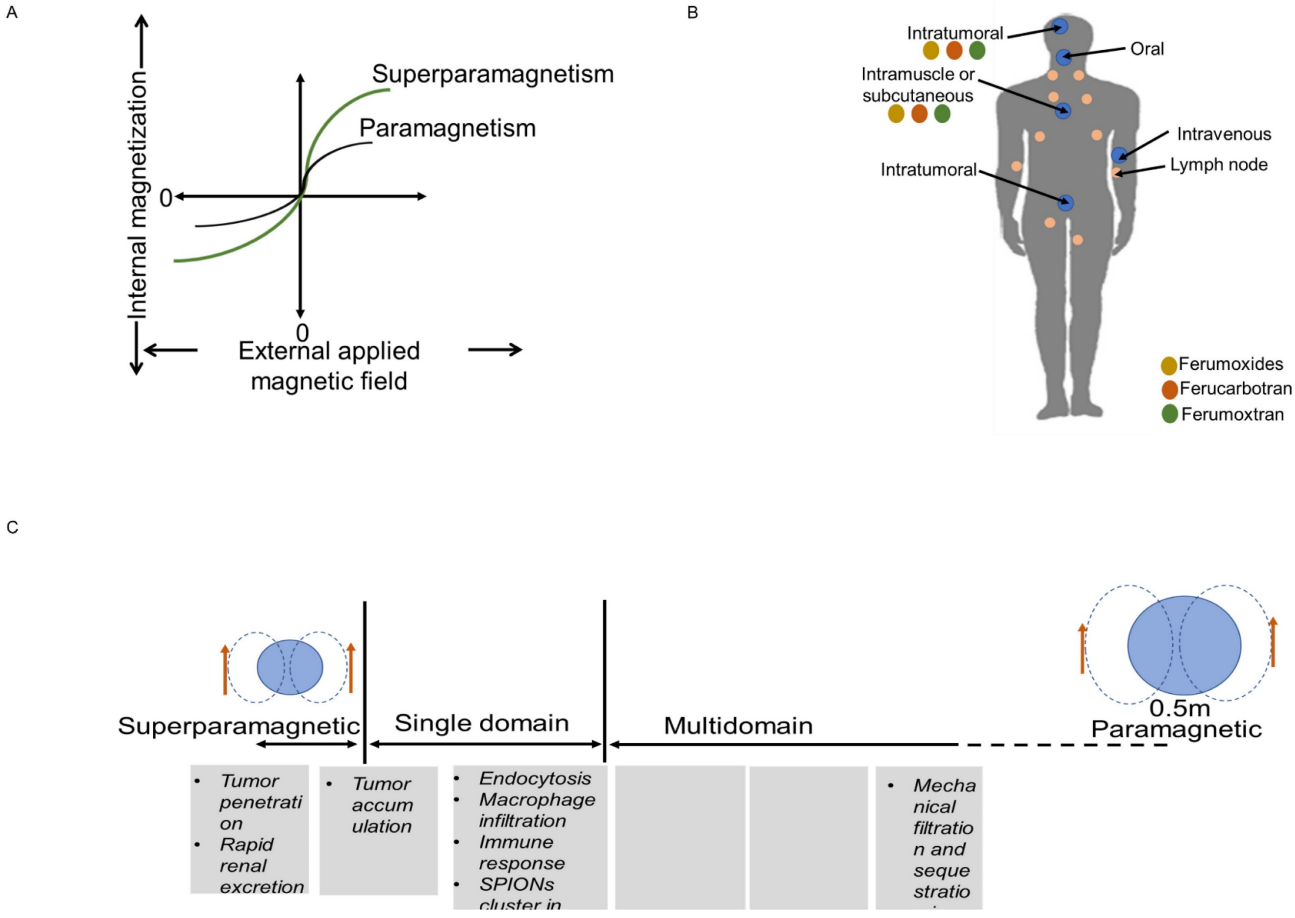
Magnetic behavior of IONPs (A), Route of administration (B) and Biological distribution of different sizes of IONPs (C).

MNPs are far more sensitive to external magnetic fields than bulk materials because there are more electrons in MNPs that spin in the same direction. Furthermore, magnetic field strength varies with size. For instance, IONPs with a diameter of less than 20 nm have a single domain of electrons that spin in the same direction, but IONPs larger than 20 nm contain numerous domains of electrons that spin in the opposite directions. Consequently, compared to other paramagnetic materials, IONPs have a stronger magnetic liability to external magnetic field. When the external magnetic field is eliminated, IONPs also have the capability of demagnetization, which is crucial for biomedical applications. Since IONPs produce localized heterogeneities in presence of external magnetic field, this affects the images (Figure 2A) 32. On T2-weighted images, this reduces their signal, giving the IONPs to look dark; however, smaller IONPs can also create positive contrast on T1-weighted images, rendering them brighter 28. Higher contrasts for MRI are achieved by producing nanocrystals with diameters of 4, 6, 9, and 12 nm and subjecting them to a 1.5 T magnetic field, respectively. The creation of better IONPs for T1 contrast involves increasing the ratio of maghemite (Fe_2_O_3_), which has a lower magnetization, to magnetite (Fe_3_O_4_) 26. Of note, maghemite and magnetite are two nanocrystals, which are widely used in biomedical field. At ambient temperature, the molecular structure of magnetite (Fe_3_O_4_) allows electrons to jump between Fe^2+^ and Fe^3+^ ions in the octahedral sites, making it an important class of half-metallic substances. Their small size also allows them to display useful distribution behavior throughout the body. Along with possessing a superior T1 contrast and having a non-toxic nature, IONPs also provide details on the mechanisms of disease progression and therapeutic efficiency of drugs than the traditional Gd based contrast agents Gd-DPTA and Gd-DOTA 32,33.

While size are important factor to the magnetic properties and their use as contrast agents, size can also affect cellular uptake, biodistribution, mode of endocytosis and efficiency of the particle in the endocytic pathway 30.

The route to uptake IONPs in human and animals is oral, local injection or intravenous injection (I.V.) (Figure 2B-C). After I.V. injection into humans and animals, free IONPs are readily taken up by the reticuloendothelial system (RES) in the liver and by macrophages. The reticuloendothelial system (RES) is prone to quickly detecting large particles (>50 nm), which are subsequently removed from the blood stream. The advantage of having a larger diameter is that it increases the negative contrast provided in T2-weighted images. But this can change the even biodistribution throughout the body. Since macrophages are one of the immune cells that take part in the inflammation process, uptake of IONPs by macrophages enable detection of inflammation by MRI. IONPs with size range between 10 and 30 nm can diffuse across plasma membrane and enter cells through membrane channels. Smaller IONPs (~5nm) are quickly eliminated from the body through the renal system. Larger IONPs cannot be removed with this process, they remain in the body for a longer period of time, preventing longitudinal re-imaging 34. IONPs larger than 200 nm can activate the complement system, unless their surface is engineered 32.

Biomedical application that requires tissue penetration can be used with IONPs smaller than 100 nm, however, those closer to 5 nm are better at penetrating tumors. Size also has an impact on cellular functions such as positioning inside the cell, uptake of more particles, and molecule binding affinity 32,34.

**Figure 3:**
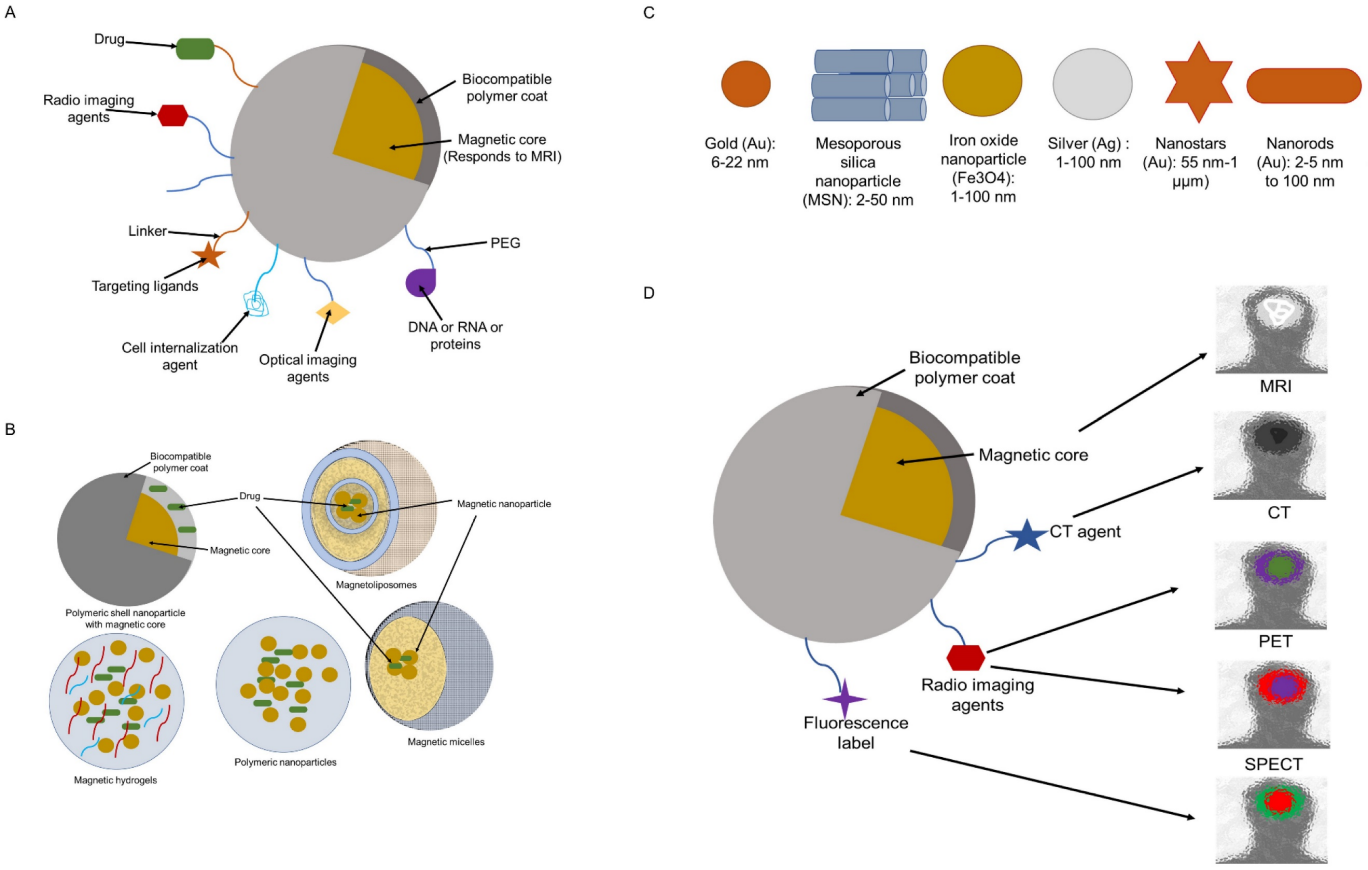
Multifunctional SPION (A), Different biocompatible coatings (B), Inorganic nanoparticles (C) and, SPIONs used is multimodal imaging (D)

A typical IONP system is made up of an (i) polymer shell that helps to maintain stability and biocompatibility, (ii) a magnetic core that enhances contrast, and (iii) often surface-bound or embedding molecules that give target-specific capability and/or multi-functionality. Moreover, therapeutic payloads may be added to a multifunctional IONP, that acts as both a therapeutic carrier and an imaging agent (Figure 3A) 35.

### Shape

The shape of NP influences both its ability to internalize and its interaction with membrane receptors. The shape of Fe_2_O_3_ nanoparticles is known to be affected by several variables, such as the catalysts and reaction conditions. Magnetite and maghemite have spherical shape and their homogenous surface area enable the coating and conjugation of therapeutic compounds or targeted ligands. Shape of IONPs is directly related to cell toxicity. As compared to nanorods and colloidal nanocrystal, the cellular toxicity of IONPs with the shapes of nanobead, nanoworm, and nanosphere are higher 30,32.

### Charge

Surface charge is crucial to the biodistribution of nanoparticles. High surface charge (positive or negative) increases phagocytosis while neutral particles have the lowest interactions with plasma proteins (and less opsonization), which prolongs circulation time. Strongly negatively charged particles are picked up by the hepatic clearance system, whereas positively charged particles are pulled by proteins and the immune system and are quickly removed from the systemic circulation (within a few minutes). Since there are only a few hydroxyl groups on the surface of bare iron oxide nanoparticles, they are typically negatively charged. Most of the time, solid particles in an aqueous electrolyte solution gain a surface charge. Certain types of nanoparticles (such as iron oxide and gold) are typically given this surface charge to increase their stability and avoid aggregation in aqueous solution via electrostatic repulsion. Different polymers (discussed below) may functionalize MNPs to add charged groups that not only make the particles more water soluble and prevent them from aggregating, but also change how they interact with blood proteins 32.

### Colloidal Stability

One of the key requirements for nanoparticles in biomedical applications is colloidal stability. Due to hydrophobic interactions, naked iron oxide particles are unstable in aqueous conditions and tend to aggregate. Aggregation increases size of the nanoparticles significantly and decreases the effectiveness of the particles. IONPs' colloidal stability is enhanced in a wide pH range and in the presence of a large number of electrolytes by the surface modification of IONPs by grafting stabilizing agents, natural or synthetic polymers that makes these NPs biocompatible 32.

### Drug loading

The essential prerequisite during drug loading is that its functionality should not be affected. Additionally, the drug should be released by these drug-loaded nanoparticles at the proper location and rate. It is possible to achieve drug loading by coencapsulating drug molecules with magnetic particles inside the coating material envelope or by conjugating the therapeutic molecules on the surface of IONPs. Several strategies have been explored for conjugating drugs or targeting ligands on the surface of IONPs 30.

Conjugation can be performed by cleavable covalent linkages and by means of physical interactions. In covalent linking techniques, the therapeutic agent or the target molecule is linked directly to, for instance, the amino or hydroxyl functional groups found on the surface of polymer-coated IONPs. The drug can also be attached to the particle using linker groups such as iodoacetyls, maleimides, and the dual-purpose linker pyridyl disulfide. This strategy results in more precise interconnections as well as improved loading capacity, safeguarding the drug's functionality and efficacy. Utilizing linkers has the additional benefit of having milder binding conditions, making it appropriate for pharmaceuticals such therapeutic peptides and proteins that are susceptible to oxidative destruction 30.

Drug molecules can couple on the surfaces of IONPs by physical interactions also. These can be electrostatic contacts, hydrophobic/hydrophilic interactions, and affinity interactions. Positively charged DNA interacts electrostatically with IONPs coated in polyethylenimine (PEI), a cationic polymer, confirming their suitability as transfection agents 30.

Similar electrostatic interactions allow negatively charged functional groups on dextran-coated IONPs to bind with peptide oligomers. Due to hydrophobic interactions, lipophilic drugs can readily connect to IONPs coated in hydrophobic polymers. The drug is then released as the coating breaks down. The bioconjugation of targeted agents or drugs with IONPs can also make use of affinity interactions, such as streptavidin-biotin interactions. Affinity interactions, as opposed to electrostatic and hydrophobic contacts, provide the most durable noncovalent linkages that are largely unaffected by environmental factors such variations in pH and medium ionic strength 30.

Another method of delivering a medicine to the target region involves loading drug molecules and iron oxide nanoparticles within the coated material. This method offers promising solutions to issues like instability and low entrapment efficiency. Magnetoliposomes (MLPs) (Figure 3B) are a novel class of nanocomposites with improved stability and magnetic characteristics and ensure high entrapment efficiency. MLPs are SPIONs, encapsulated within liposomes 36-38. The size, charge, and surface features of liposomes can be easily altered for various in vivo delivery applications by merely introducing new components into the lipid mixture prior to liposome synthesis or by altering the preparation techniques. To effectively stabilize and prolong the residence period in blood circulation, hydrophilic polymers, such as polyethylene glycol (PEG), can be incorporated into the liposome surface. Additionally, targeting ligands (such as peptides or antibodies) can be affixed to liposomes to improve their selectivity for the intended target tissues 37. Furthermore, lipophilic drugs can be solubilized within the lipid bilayer while hydrophilic drugs can be trapped in the central aqueous core of the liposomes. In either instance, the drug is trapped within the liposome until the nanoparticle reaches the target site, such as a tumor 39. Thus, liposomes are flexible nanoparticles with low toxicity that can be made from a variety of natural and synthetic lipids with biological origin and used as drug carriers to lessen the toxicity of powerful drugs such as doxorubicin, platinum anticancer drugs, cathepsin protease inhibitor, irinotecan, vinorelbine, parthenolide, and diclofenac sodium in non-cancerous cells and tissues. Paclitaxel, which is insoluble in water and challenging to alter, can be successfully placed in liposomes to enhance the therapeutic effect 40. Liposomes are also an excellent example of a nano-delivery system that may deliver both drug and imaging substances. Better biocompatibility, biodegradability, less toxicity, and the potential to modify size and surface properties are all advantages of liposomes. These features set liposomes unique from other delivery systems 40. MLPs, which are spherical nanostructures with sizes between 100 and 150 nm, are made of liposomes and MNPs. Nevertheless, their ultimate size depends on the liposome synthesis method. MLP vesicles made of magnetic nanoparticles are encased in a bilayer of phospholipids 37,38. The hydrophobic areas that can be exploited for drug encapsulation are retained by theMLPs. There are various benefits to pharmaceutical grade saturating IONPs within the phospholipid envelope. Firstly, liposomes with magnetic particles offer quick and cheap surface alterations that make it possible to focus them on a particular tissue 30. Secondly, due to their higher entrapment effectiveness, MLPs with high IONP concentrations offer the best magnetic responsiveness, and their nanoscale allows for the use of the enhanced permeability and retention (EPR) effect for tumor targeting. In addition, a bigger amount of both hydrophilic as well as hydrophobic medication can be placed within the liposomal framework together with IONPs. Thirdly, IONPs' biocompatibility is further enhanced by liposomal encapsulation. Fourthly, the liposomal barrier shields medications from the environment's deteriorating effects 30.

There are two ways to load IONPs into the liposomes: either by directly precipitating the IONPs within the hydrophilic core of the liposomes, which results in highly uniform nanoparticles with sizes around 15 nm, or by incubating previously prepared liposomes and IONPs under the influence of an external force. When MLPs are exposed to a strong permanent magnetic field over the target tissue, magnetic particles within the bilayer are aligned, leading the MLPs to collect, fuse, and release the drug precisely where it is required 30.

Another type of nanocomposites that are ideally suited for MRI imaging of cell migration and trafficking are magnetodendrimers. Iron oxide nanoparticle solutions are frequently coated with and stabilized with carboxylated polyamidoamine dendrimers. In general, the oxidation of Fe (II) results in very stable and soluble IONPs with dendrimers at high temperatures and pH levels. Dendronized iron oxide nanoparticles have been synthesized for multimodal imaging. A special potential for labelling with a fluorescent dye exists with IONPs coated with dendrimers that have hydrodynamic sizes smaller than 100 nm and either carboxylate or ammonium groups at the periphery. It has been shown that magnetic resonance and fluorescence imaging are both feasible simultaneously 30.

### Targeting

The targeting of iron oxide contrast agents at the specific site of interest is accomplished by the conjugation of ligands to functional groups on the surface of the NPs (Figure 3A). Due to the high surface area to volume ratio, it is possible to link numerous copies of a ligand to a single NP. The binding affinity of NP is greatly increased by this multivalent targeting, enabling each particle to attach to several copies of receptors produced on cell surfaces. targeting ligands include antibodies or their immune-specific fragment F (ab), aptamers, peptides, or small molecule peptideomimetics emerging from phage display or small-molecule screens 41. Iron oxide nanoparticles are functionalized with reactive surface groups such as carboxylic acid or amine surface groups. These enable covalent conjugation of protein, antibodies, or small peptide ligands on NPs. Carboxylic acid functionalized nanoparticles require prior activation with either carbodiimide, most commonly 1-ethyl-3-(3-dimethylaminopropyl) (EDC), or a combination of EDC and N-hydroxyl succinimide ester (NHS). Amine functionalized nanoparticles can be directly coupled to reactive ligands through reductive amination of aldehydes. To allow the conjugation of sulfhydryl-containing ligands, such as those found in peptides and antibodies, the surface of amine-functionalized nanoparticles can also be changed by coupling cross-linking agents, such as succinimidyl iodoacetate (SIA), N-succinimidyl-3-(2-pyridyldithio) propionate (SPDP), or succinimidyl-4-(N-maleimidomethyl) cyclohexane-1-carboxylate (SMCC) 41,42.

### Coating

IONPs can be synthesized by a variety of synthetic processes, such as flow injection synthesis, sol-gel synthesis, sonochemical reactions, hydrothermal reactions, hydrolysis and thermolysis of precursors, and electrospray synthesis. Surface complexing agents must be applied to IONPs. After modification, it is essential to prevent nanoparticle aggregation, reduce toxicity, and achieve altered pharmacokinetics and biodistribution. It is often preferable to select a surface coating containing functional groups to assist in the attachment of selected ligands 34.

For clinical use, IONPs are coated with a biocompatible material (Figure 3B) 28. Uncoated IONPs with a size of 16 nm exhibit a considerable tendency to aggregate or precipitate at concentrations of 3 mg/mL in saline. Numerous studies have been conducted to reduce cytotoxicity by developing coatings that can prevent precipitation and aggregation. Because the coating can prevent the release of iron ions and regulate how the nanoparticles interact with their biological surroundings, it has a substantial impact on the toxicity of the nanoparticles (cells, proteins, etc.). The method of cellular absorption and the destiny of IONPs inside the cell depend on the size and hydrophilicity of the surface-modifying agents. Particles with sizes more than 100 nm begin to be strongly phagocytosed, whilst those less than 30 nm are swiftly picked up by pinocytosis. A higher hydrophilicity lowers pinocytosis by preventing a particle from passing through the hydrophobic, lipid bilayer of the cells 42.

Several substances, such as dextran, polyethylene glycol (PEG), polyvinyl alcohol (PVA), chitosan, and other polymers, may be used to construct the coating. These hydrophilic molecules on the surface of IONPs stabilize these when surrounded by water. The strong magnetic dipole-dipole interactions between the iron oxide cores are interfered with by the high molecular weight coatings made of PEG, PVA, and dextran, which prevent IONPs from aggregating. Another advantage of coating IONPs is that longer relaxation times lead to better imaging results. Additionally, it hinders or prevents the release of Fe^2+^ when exposed to lysosomes, thereby increases the long shelf-lives of IONPs. The best biocompatible coating disintegrate into particles smaller than 5 nm, minimizing cytotoxicity during renal excretion 43.

Dextran-coated NP exhibits great biocompatibility and no cytotoxicity or inflammation at dosages of 11.3 ug/ml. The iron from the core of IONPs accesses the body's iron stores before becoming a component of hemoglobin, but dextran is slowly degraded and 89% of it is excreted by urine within 56 days, further showing the safety of utilizing dextran-coated IONPs. The flexibility of dextran-coated IONPs is demonstrated by their potential to target a variety of physiological systems. Dextran-coated IONPs can be functionalized to image human lymphocytes, myocardial infarction, monitor T cells, and for the early identification of chronic allograft rejection in addition to imaging macrophages in arthritic joints 25.

Another typical coating used to lessen interactions with cells or proteins and neutralize the IONPs' surface is polyethylene glycol (PEG). PEG has the highest solubility and is hydrophilic (HD) and zwitterionic. PEG has good biocompatibility and do not have potential toxic groups. After pegylation, PEG-coated IONPs reduce to 10 to 15 nm from 40 to 50 nm in size. This depends on the molecular weight of the PEG used. Pegylated IONPs can significantly preserve T2 and T2* relaxation-based contrast in MR imaging. Deoxygenated hemoglobin, methemoglobin, or hemosiderin lesions in tissues can be detected using T2* weighted imaging. This pegylation effectively reduces cytotoxicity up to 1 mg/ml in contrast to uncoated IONPs. Coating also increased IONP size as well as blood circulation. There were noticeable effects on biodistribution and blood half-life. IONPs can be joined together within a single coating to produce PEG-IONP nanoclusters which has greater MRI contrast. Pegylation of IONPs shields them from enzymes and antibodies that may induce degradation, secretion, and clearance from the immune system. Interestingly, PEG-coated IONPs can be still recognized by macrophages or other immune cells. Stealth coating by PEG also leads to platelet membrane cloaking, which reduces cell uptake and complement activation 25,33.

PVA (polyvinyl alcohol) is another highly biocompatible coating agent. PVA IONPs are scarcely captured by the cells, if there is no magnetic field applied. PVA coated IONPs are highly beneficial as they are able to prevent clumping inside narrow vessels of 7-8 µM in diameter 33.

Other coating agent for IONPs, such as silica has also demonstrated great capabilities due to the biocompatibility, low cost, and tolerance of a wide range of pHs. The protective effects of silica have been demonstrated by a fewer number of Fe^3+^ ions released from IONPs and a slower metabolism and excretion rate of IONPs, while at the same time not reducing the rate of uptake. Porous silica gel encapsulations provide intimate contact between the water molecules and the iron oxide core for enhancement of MRI contrast while eliminating the toxicity of IONPs and providing a framework for attaching many different types of ligands to IONPs such as ^64^Cu and ^111^In 35,43. Table 1 shows some of the coating material used with IONPs and their clinical use.

Once synthesized, the usefulness of IONPs for MR imaging are investigated. Nuclear magnetic resonance (NMR) spectroscopy and MR scanners are used to study the longitudinal (T1), transverse (T2), and effective transverse (T2*) relaxation times and their corresponding relaxation rates R1, R2, and R2* which are the inverses of T1, T2, and T2*, respectively. The corresponding relaxivities (r1 and r2) are calculated by graphing the variations in R1 and R2 of MRI contrast agents as a function of their concentration and are commonly represented in mM-1s-1. The longitudinal and transverse relaxivities of iron oxide nanoparticles are influenced by various factors such as the IONPs' core size, shape, hydrodynamic diameter, size distribution, nanoparticles coating, and aggregation. R1 is largely unchanged for particles larger than around 8 nm, whereas r2 normally increases linearly as the particle core size increases from 4-5 to 18 nm. Because IONP are uniformly scattered in water and protons diffuse between the magnetic cores, the influence of hydrodynamic diameter and aggregation on relaxivities shows that r2 gradually increases with hydrodynamic diameter (up to about 80 nm) for tiny clusters. If a huge cluster is larger than 90 nm in size, r2 decreases with increasing size as a result of a decreased proton exchange rate driven by the reduced accessibility of the surface. The strength of the applied magnetic field, temperature, and the substance in which the contrast agent is disseminated all affect relaxivity values in addition to the characteristics of the contrast agent itself 31,32.

IONPs, can also undergo various surface modifications (e.g., the addition of a fluorescent label), to be used for multimodal imaging (Figure 3B-D) 44. IONPs can be used in combination with NIRF (Near Infrared Fluorescence) using the dye Cy5.5 (Cyanine 5.5)-SPION system, or these can also be used with CT (Computed Tomography), PET (Positron Emission Tomography), and SPECT (Single Photon Emission Tomography) agents. The core-shell designed with two separate imaging modalities, increases the sensitivity and reliability of diagnosis. The following modifications can be done:

### MR with optical imaging

In this case, the IONP core might either be directly linked to the fluorophores or coated with a biocompatible shell that is functionalized with a fluorescent probe (such as dextran, polyethylene glycol, silica, etc.). Rhodamine B, fluorescein isothiocyanate, and Cy5 are the three most widely utilized optical agents. This hybrid probe simultaneously possesses MRI resolution and optical imaging sensitivity 25. MLPs are good example of nano-delivery system for this type of multimodal construct. Due to their extensive diversity in lipid types and lipid conjugation, MLs can be designed to function as a multimodality contrast agent. The coatings, together with an intuitive synthesis process offers MLs for a wide range of intriguing applications. Bimodal contrast agents for MRI/optical imaging can be developed by labeling ML lipid bilayers using various fluorophores. The synthesis process can be simply modified to add fluorescently tagged lipid conjugates into any of the ML layers. An example of such construct is annexin A5-functionalized MLs which were developed as bimodal contrast agents for the detection of apoptosis in T-lymphoma cell line through both fluorescence and MRI 37. Using these bimodal MLs, it was possible to assess the particles' in vivo biodistribution and pharmacokinetics as well as the impact of PEGylation on mouse tissue 45. In a separate study, the interaction of MLs with human adenocarcinoma prostatic cell line PC3 was investigated in vitro by the addition of fluorescent probes.

The findings suggested that it was possible to determine that PEGylated MLs strongly interact with PC3 cancer cells when applied as bimodal contrast agents, which were detected by the two complimentary procedures, fluorescence microscopy, and magnetophoresis 46.

### MR with PET imaging

PET is extremely sensitive imaging technique that creates images utilizing signals from radioactive tracer molecules (^11^C, ^18^F, ^64^Cu, ^68^Ga, and ^124^I), but has low resolution. If a radionuclide is grafted onto an SPION that is polymer coated, this offers high-resolution, phenomenal, quantitative approach with no penetration limit 25.

### MR with CT imaging

CT is a prominent technique for medical imaging that creates images from X-rays and have been computer-processed. In contrast to an MRI, CT produce images from many different organs with great temporal resolution, but it is limited in its ability to image soft tissue because of its low sensitivity. The combination of these two modes could therefore mitigate their limitations. Iodine, the most common CT imaging agent, and noble metals (gold and silver) are employed for this purpose. These substances may either be directly applied to the surface of IONP or applied as a coating, or they might be regarded as heterostructured nanoparticles 26.

### MR with US imaging

Real-time imaging using diagnostic sonography is affordable, extremely safe, and builds images based on the varying rates at which ultrasound waves move through various tissues. Due to the penetration restriction, it could only be used for superficial and echogenic tissues. Strong contrast is obtained in MRI and ultrasound (US) by the use of microbubbles as US contrast agents that are functionalized with SPION 28.

## Application of iron oxide nanoparticles in disease diagnosis and theranostics

SPIONS have drawn significant attention in bioscience research since the first report in the 1980s. In the 1990s, SPIONs were first utilized for diagnostics 47,48. However, only a small number of SPIONs produced by pharmaceutical companies achieved several rounds of clinical trials and some of them received approval by regulatory bodies 48,49. Resovist® (Ferucarbotran, Resovist®, Bayer Healthcare, Germany) and Feridex® (Ferumoxides, Feridex® IV, Berlex Laboratories, Endorem®, France) have each gained regulatory approval in the United States, Europe, and Japan, respectively. Feridex® (Endorem®) has been taken off the market due to a lack of consumers, and Resovist® is currently only available in a small number of countries, including Japan (Resovist®; FUJIFILM RI Farma Co., Ltd., Kyobashi, Tokyo, Japan). NC100150 (Clariscan®, Nycomed, Norway) and (VSOP C184, Ferropharm, Germany) were produced and entered clinical testing for MR angiography and blood pool imaging, respectively, they were not approved by the regulatory body. Ferumoxsil (Lumirem®, or GastroMARK®) with a silica coating SPION received regulatory approval, but was also removed from the market for lack of demand. Ferumoxytol (Feraheme®, AMAG Pharmaceuticals, Cambridge, MA) which has been approved for the treatment of iron deficiency, has been withdrawn from Europe to be used as a contrast agent 50-52. Other than biomedical applications, IONPs are being used extensively in a variety of industries, including the agricultural and food sector, environmental remediation, energy, defense and aerospace, construction, automotive, textiles, and electronics 49,53. Since this review article is on the application of IONPs along with MRI and personalized medicine, therefore, we will limit our discussion in relation to disease diagnosis and theranostics applications of SPIONs.

### Application of IONPs in Disease Diagnosis

#### Cancer

Cancer is one of the major causes of death worldwide 54. MRI has been used in the screening of breast, liver, lung and colon cancer screening programs 55,56.

Lymph node staging is essential for identifying cancer types and selecting the best treatment plans. Knowing how many lymph nodes have been affected by metastatic colonization and how many lymph nodes are affected might assist anticipate how long a patient will survive. The staging of lymph nodes is frequently carried out by sentinel lymph node imaging or by surgical dissection, followed by histological analysis of a few nearby lymph nodes draining the original tumor location. Standard non-invasive lymph node staging by size has drawbacks, including a low detection rate for extremely small metastases and inaccurate positive assessments of swollen, inflamed lymph nodes as metastases. On the other hand, dissection surgery is invasive and frequently difficult to enforce. It only applies to a specific region surrounding the primary tumor, which may cause it to miss distant lymph node metastases that manifest themselves most often in the pararectal and internal iliac regions, underestimating the presence of local lymph node metastases. Furthermore, it is usually impossible to find very small lymph nodes. Ultrasound (US), computed tomography (CT), and non-contrast-enhanced MRI are non-invasive methods for assessing lymph node status. These methods are widely employed in clinical settings to classify lymph nodes based on changes in size and morphology, but they are less accurate, specific, and sensitive than IONP-enhanced MR imaging. IONPs with hydrodynamic diameter of 20 to 30 nm has a prolonged circulation period, making it useful for imaging lymphography, inflammation, and blood pools. When administered intravenously, IONPs circulate in the bloodstream, partially extravasate into tissues over time, and then are eventually removed by lymph system and accumulate in macrophages in lymph nodes. This causes the signal intensity in T2 and T2*-weighted MR imaging to diminish and/or causes the signal intensity in T1-weighted imaging to increase. Reduced nodal macrophage concentration and compromised lymph node function are results of metastatic manifestation in lymph nodes. Additionally, metastasis may cause macrophage functioning to reduce and phagocytic activity to decrease. Lower IONP uptake and brighter malignant lymph nodes can be seen as a result on T2/T2*-weighted imaging 16.

In clinical trials, SPION-based ferumoxytol (Feraheme®, USA) and ferumoxtran (Combidex®, USA, Sinerem®, EU) were used for lymph node imaging to investigate metastatic colonization. Ferumoxtran is used to detect macrophage infiltration. The use of ferumoxtran compared to non-contrast-enhanced MR imaging showed noticeably improved sensitivity for lymph node characterization in patients with prostate cancer, in whom metastases detection with positron emission tomography (PET) is challenging 24.

MR lymphography with ferumoxtran enhancement showed high sensitivity in detecting lymph node metastases, although it has not yet been used in clinical settings due to conflicting results. However, USPIO-based contrast-enhanced MRI proofed to be useful for disease staging and therapy selection for breast, head and neck, esophageal, and pelvic cancer, in which lymph node colonization occurs 16.

IONPs have also been used a lot to visualize primary liver lesions, such as hepatocellular carcinoma (HCC) and liver metastases, because of their high liver absorption by the macrophages of the mononuclear phagocytic system (MPS) 55.

In addition to its use in lymph node staging, SPION-based contrast agents have also been used on the characterization of liver function using MRI during radiation therapy. The hepatic Kupffer cells (KC) in the liver phagocytose super-paramagnetic iron oxide nanoparticles (SPIONs), which shorten MRI signals in the functional liver parenchyma (FLP) volume containing KCs. Malignant tumors without KCs, however, show little signal change, leading to an increase in hepatic heterogeneity. To differentiate FLP and non-FLP, research was conducted to examine whether SPIONs enhance liver heterogeneity on R2*-MRI (i.e., tumor, hepatic vessels, liver fibrosis and scarring associated with hepatic cirrhosis, prior liver-directed therapies, or hepatic resection). Liver heterogeneity was enhanced during two MRI sessions with and without an intravenous SPION injection and the volume of FLP was recognized in auto-contouring system. This is a promising means of obtaining more precise liver function and tumor characterizations during radiation treatment planning 57.

Table 2 shows some of the MRI biomarkers that are validated and used in clinics for cancer.

#### Liver disease

Kupffer cells are crucial to the regeneration of the liver. There is no clinically safe and reliable way to assess Kupffer cell function, even though it is linked to poor outcomes. After injecting SPIONs, which are captured by Kupffer cells, magnetic resonance imaging was used to assess Kupffer cell function in healthy liver or in obstructive jaundice. Every 4 minutes for 60 minutes following SPION injection, the liver parenchyma's T1-weighted signal intensity was analyzed. Following the buildup of iron in the liver in both groups, the signal intensity levels gradually dropped. Both groups experienced a similar and considerable rise in serum iron levels. However, between 8 and 20 minutes after superparamagnetic iron oxide injection, the values of relative enhancement, or the percentage of signal intensity from pre-contrast to postcontrast, were significantly higher in the group with obstructive jaundice than in the control, indicating Kupffer cell dysfunction in obstructive jaundiced liver. These findings suggested that Kupffer cell function in individuals with obstructive jaundice might be evaluated using chronological magnetic resonance imaging with SPIONs 58.

#### Neuroinflammation

MRI allows visualization of neuroinflammation in vivo. SPIONs are phagocytosed by hematogenous macrophages upon systemic application into the circulation and allow, due to their paramagnetic effect, in vivo tracking by MRI of infiltration to the CNS in experimental CNS disorders, and in multiple sclerosis (MS) and stroke. Targeting of inflammatory, activation-dependent enzymes such as myeloperoxidase or immune function molecules by MR contrast agents represents a molecular approach to visualize critical steps of lesion development caused by neuroinflammation 58.

The dynamics of neuroinflammation can be better understood with the help of cellular and targeted molecular imaging. Given the crucial role that inflammation plays in the pathogenesis of many nervous system illnesses, it will also be essential for recovery processes like peripheral nerve regeneration. Prior to clearance inside the reticuloendothelial system (RES) of the liver, spleen, and lymph nodes, monocytes predominantly phagocytose SPIONs. Due to the superparamagnetic properties of iron oxide, which cause a signal loss on T2-weighted images and/or strong contrast on T1-weighted images, these iron-oxide-laden macrophages become detectable in MRI upon acute migration into the damaged neurological system. In vivo imaging of cellular inflammation during Wallerian degeneration, experimental autoimmune neuritis and encephalomyelitis, stroke in rodents, as well as stroke and MS in patients, has been made possible with SPIONs. Molecular MRI can be expanded by coupling antibodies to SPIONs, and it has been used to determine the cell adhesion molecules that mediate inflammation 58.

MRI has been utilized rather frequently in the diagnosis and staging of multiple sclerosis (MS) to assess the blood-brain barrier's (BBB) integrity and to identify inflammatory lesions in the brain. The ability of iron oxide nanoparticles to visualize monocyte/macrophage infiltration, which coincides with ongoing demyelination and is a hallmark of MS disease progression, in this context is a significant benefit over gadolinium-based contrast agents. Gadolinium-based compounds visualize an increase in BBB permeability while providing relatively general and indirect imaging information on inflammation. Additionally, USPIO nanoparticles are taken by CNS macrophages and as a result, reveal details about macrophage content and colonization. Therefore, compared to gadolinium alone, the use of USPIO in conjunction with gadolinium chelates in MS patients enables detection of more active lesions. Higher lesion detection accuracy has been attained by combining the two agents, which enhances the ability to identify patients with active MS. Lesions that are visible on both gadolinium- and USPIO-enhanced images have been found to be larger and more aggressive in their ability to cause tissue damage than lesions that are only enhanced by one of the two agents. Future clinical use as a marker for MS disease activity may involve this dual imaging agent MRI technique 16.

#### Autoimmune disease

The immune system comprises of small molecules, soluble proteins (such as antibodies, cytokines, chemokines, complement proteins, etc.), cell surface proteins (such as receptors, adhesion molecules, etc.), and immune cells (macrophages, monocytes, dendritic cells, natural killer cells, T cells and B cells). This intricate system functions to eliminate invasive pathogens, remove damaged cells and debris, and monitor against cancerous cells. Components of the immune system are broadly divided into innate and adaptive immune responses. Components of the innate immune response are similar among individuals and are generally targeted by conserved molecules expressed by pathogens. Conversely, when the host is exposed to foreign molecules, the adaptive immune responses are transformed. As a result, the range of adaptive immune system receptors varies between individuals and changes over time 59,60. Recognizing invading pathogens from host cells, which allows the immune system to be instantly activated, is a crucial function of both the innate and adaptive arms of the immune system. Immune cells, however, also respond locally. This can lead to inflammation and tissue damage in a variety of situations. The immune system affects the host by causing inflammation. For instance, in certain chronic infections, the immune system is unable to eliminate the infection, and a persistent inflammatory response can severely damage tissue, resulting in autoimmune disease. In autoimmune diseases, the adaptive immune system responds to host epitopes as though they were foreign and this immune response can damage target organs permanently. Mutations in adaptive systems can potentially result in autoimmune diseases. Over 100 distinct autoimmune conditions exist 59,60.Patients with autoimmune diseases have a wide range of symptoms, making diagnosis difficult. Imaging can be used to characterize the disease 60.

An example of this approach is the imaging of insulitis, an autoimmune illness characterized by leukocyte infiltration and inflammatory tissue damage to pancreatic islet cells. Insulin-producing beta cells are found in the islets, and when these cells are damaged, humans develop type 1 diabetes (T1D). In order to discover T1D early, monitoring insulitis via imaging pancreatic inflammation is a good option. Using USPIO based on ferumoxtran was used to monitor leukocyte (macrophage) infiltration. Patients with insulitis displayed an inflammation-induced modification in their pancreatic microvasculature, known as increased vascular leakiness. Circulating USPIO extravasates into the inflammatory tissue and is absorbed by macrophages, resulting in USPIO enrichment that may be observed on MRI. This enables the distinction to be made between diabetics and healthy people at the early stage of the disease 16,61.

#### Stroke and cardiovascular disease

Cerebral stroke continue to be one of the leading causes of fatalities or serious disabilities in developed nation. Brain inflammation is a potentially significant pathomechanism of ischemic brain damage, in a subacute time frame following stroke onset. The mononuclear phagocyte system, which includes resident microglia/brain macrophages as well as hematogenous macrophages, predominates in postischemic inflammation. Hematogenous macrophage infiltration is delayed, but microglia activation happens quickly minutes after the assault. The exact time window differs between studies and models. Earlier efforts at treating ischemic stroke with anti-inflammatory medication in a clinical environment have been unsuccessful. Therefore, methods for the noninvasive detection of inflammation have great potential for the development of novel therapeutics 62. USPION that are macrophage specific and can be administered intravenously, showed differences in signal intensity in MRI. This was also observed in patients. Three out of the nine patients who were studied showed parenchymal SPION enhancement, which was primarily visible on T1-weighted spin-echo imaging. Variations of the SPION-dependent signal alterations reflected the distinctive patterns of hematogenous macrophage infiltration in various lesion types. In order to more precisely target anti-inflammatory therapy in stroke patients, SPION enhanced MRI may be useful 63.

Studies of both experimental ischemia and clinical stroke have shown that SPION-laden macrophages can generate the usual signal alterations in the MRI of ischemic brain parenchyma. The involvement of macrophages in the formation of ischemic lesions may thus be addressed scientifically and clinically using SPION-enhanced MRI 34.

SPIONs can also be used to identify vascular inflammation in atherosclerotic plaque. Numerous studies have demonstrated the viability of imaging rabbit atherosclerotic plaques using SPIONs for in vivo imaging by MRI 58. An atherosclerotic plaque rupture can cause an artery to become blocked, causing a myocardial infarction, stroke, or renal artery stenosis; for this reason, such events must be thoroughly and meticulously monitored. While no effect was shown in the control animals, the uptake of SPIONs in the vessel wall of atherosclerotic rabbits resulted in a significant decrease of focal signal in gradient echo pictures. A team of researchers created a new contrast agent for molecular diagnostics of atherosclerotic plaque. The peptide was grafted onto the USPIO after being chosen by phage display. USPIO-PEG-R832 is a novel nano system that was initially tested on HUVEC (Human Umbilical Vein Endothelial Cells) and then by MRI on Apo E-/- mice 58.

P904, another sort of vectorized SPIONs, was investigated in a rabbit model of induced aortic atherosclerosis. Following the administration of P904 and a reference standard, ferumoxtran-10, in hyperlipidemic New Zealand white rabbits, in vivo angiography and T2*-weighted plaque MR imaging were carried out. Plaque analysis was possible as soon as 24 hours following P904 injection using in vivo MR imaging. After administering P904, the authors found that vessel wall area increased by susceptibility artifacts by 27.75% on day 2 and by 38.81% on day 3, as opposed to 44.5% on day 7 and 34.8% on day 10 after administering ferumoxtran-10. These artifacts were correlated with the intraplaque iron uptake in the respective histological slices. Thus, SPIONs can be employed in order to identify vascular inflammation in atherosclerotic plaque 34,58.

Atherosclerosis can also cause aneurysms. Aneurysm is bulging in blood vessel. Because of their capability to secrete proteinases, macrophages play a central role in the growth and rupture of aneurysms. Noninvasive imaging of macrophages, therefore, may yield valuable information about the pathogenesis of aneurysm disease. Studies were conducted on the uptake of the macrophage-specific contrast agent SPIONs in the walls of aneurysms and normal sized aortas. It was observed that SPION uptake is limited or absent in the wall of normal-sized aortas and most aneurysms. However, individual abdominal aortic aneurysms exhibit high levels of SPION uptake, indicative of extensive macrophage infiltration in the aneurysm wall. Future research should focus on the predictive value of SPION uptake for the growth and rupture of aneurysms 58.

#### SPIONs in sepsis

Sepsis, a form of systemic inflammatory response syndrome brought on by infections, is increasingly leading to critical care unit admissions and has a high fatality rate while still in the hospital. Sepsis will quickly progress into septic shock, multiple organ dysfunction syndrome (MODS), and death if medical treatment is neglected. The prevalence of sepsis is continuing to rise as a result of the advent of infections that are drug-resistant and the widespread use of immunosuppressants. Due to the acute nature of sepsis, prompt diagnosis and treatment are necessary to reduce morbidity and death and enhance the outcomes of associated therapeutic interventions. Sepsis is over one of the illnesses with the highest range of symptoms and pathogenesis, making diagnosis extremely difficult. Additionally, no established diagnostic tool has yet been created to identify the development of sepsis in a clinical setting. The rapidity and accuracy needed to provide prompt, accurate diagnoses for timely treatment are sometimes lacking in real primary diagnostic tests that base their results on the measurement of vital signs and scores. Although many biomarkers in serum have been established for the diagnosis of sepsis, the required sensitivity and reliability are still insufficient. Microbiological tests have been recommended to aid in the diagnosis of sepsis in order to evaluate the underlying illnesses. But positive bacterial growth typically takes a long time in microbiological cultures—sometimes even a few days—and only 30-40% of cases of "sepsis" are culture-positive. In other words, sepsis is rarely microbiologically verified for prompt medical care. Therefore, there is an urgent need for the development of precise techniques for early diagnosis of sepsis 64.

During the development of sepsis, a systemic inflammatory response is generally brought by the activated host immune system. In the early stages of sepsis, a lot of reactive oxygen species (ROS), such as hypochlorite ions (ClO^-^), hydroxyl radicals (OH^-^), superoxide anion radicals (O2^-^), and peroxynitrite (ONOO^-^), are produced. Sepsis typically results in heightened systemic ROS levels both in the circulation and in the affected organs, in contrast to local inflammation and infections during which ROS are produced in large quantities in certain inflamed tissues and organs. Additionally, a significant contributing factor to MODS in sepsis is the systemic overproduction of ROS. As a result, ROS can be used as an alternative and predictive biomarker for sepsis. A sensitive and timely approach to monitor systemic ROS in biological systems is also helpful for the study of sepsis and ROS biology. Reactive oxygen species (ROS), which have also been referred to as sepsis biomarkers, are produced in excess in the blood and in the affected organs during sepsis. However, it is challenging to obtain ROS tracking with an unlimited imaging depth in vivo for sepsis diagnosis because the majority of ROS produced during sepsis are excessively generated in deep tissues and organs, such as the liver and kidneys. This is due to the poor penetration and low soft tissue sensitivity of imaging technologies based on optical devices 64. To test ROS by MRI in a mouse model of lipopolysaccharide (LPS)-induced sepsis, the clinically approved gadolinium-diethylenetriamine-pentaacetic acid (Gd-DTPA) with a HA-decorated iron oxide core (SPIONs) was recently used as a contrast agent. Ultrasensitive (0.2×10-6m) ROS imaging in vivo was made possible by the limitless tissue penetration depth of SPION nanoprobes, the HA-triggered ROS breakdown mechanism, and the consequent release of Gd-DTPA 64*.*

#### Septic arthritis

It is an infection of the tissues and synovial fluid of the joint. A joint can become infected by numerous types of bacteria, viruses, and fungus 58. In a new study, an experimental rabbit model of bacterial knee infection was used to test macrophage imaging with SPIONs. All the inoculated knees displayed infectious synovitis and heavy macrophage infiltration. 24 hours after SPION administration, signal loss in these infected knees was assessed qualitatively and quantitatively on T1-weighted, T2-weighted, and T2*-weighted images, reflecting the presence of SPION-loaded macrophages in the synovium. The control knees, which displayed a normal synovium devoid of the infiltration of iron-loaded macrophages, did not exhibit any significant MR signal changes in contrast 58,65.

#### Osteomyelitis

This is bacterial infection of the bone caused by *Staphylococcus aureus* 58. Research was conducted to assess SPION enhanced (SE) MR imaging for the distinction between sterile inflammation and vertebral infectious osteomyelitis in two groups of rabbits. Unenhanced and gadolinium-enhanced fat-saturated SE T1-weighted sequences were used in the MRI tests. A second MRI scan was done 24 hours following SPION injection once end plate enhancement was seen on the T1 gadolinium-enhanced MR sequence. The results of histopathology were linked with MRI. Both groups showed a considerable increase in vertebral end plates on gadolinium-enhanced T1 sequences, with no discernible difference between them. On SPION enhanced T1 sequences, only the infection group showed a significant SNR increase, and there was a significant difference between the infection and the sterile-inflammation groups in SNR. Infected areas displayed a severe infiltration of macrophages, some of which were iron-loaded, replacing the bone marrow. In sterile inflammation, the bone marrow was replaced by inflammatory tissue that had very few macrophages. Infectious osteomyelitis and sterile vertebral inflammation can be distinguished with SPION-enhanced imaging because of the distinct distributions of macrophages in the two lesions 58.

### Potentials of SPIONS as Theranostics in diseases

Theranostic imaging is a rapidly growing medical field driven by advances in various fields of materials science, synthetic chemistry, and molecular imaging. The term "theranostics" describes the fusion of diagnosis and treatment 4,5. In the field of nanomedicine, the term theranostics is used to refer treatment where imaging agents and therapeutic molecules are co-loaded in a single nanoparticle. Iron oxide nanoparticles are inherently quite promising for theranostic applications since the surface of the nanoparticle can be engineered to achieve layered structure, producing multifunctional nanoparticles for the prevention, detection, and treatment of diseases 16,44,66. Functional SPIONs can be generated by layering iron-oxide nanoparticles which will consist of three key components. Mentioned earlier, these components include an iron oxide core that serves as an MRI contrast agent, a biocompatible coating material, and a therapeutic coating that is tailored with pharmacogenomics biomarker (Figure 3A). By carefully considering and evaluating each of the three variables in relation to the goals of the multilayer nanoparticle, the most suitable option can be obtained. This will enable targeted accumulation in the area of interest, allowing for the diagnosis of diseases and the assessment of treatment efficacy, as well as tracking drug delivery and release to the targeted tissue or cell, allowing for the delivery of personalized therapy. In contrast to their straightforward administration, the usage of SPION coated with active gene/drug molecules as therapeutic carriers may increase the drug/gene delivery into the targeted tissues. This is integrally linked to the distinctive delivery system, which in the case of SPION prevents drug accumulation in healthy regions. In the multilayered approach, drugs might be incorporated into the SPION final formulation to provide protection from two potential threats: (i) the body's defense mechanism, and (ii) an early degradation by a biocompatible coating. The most common component used for the manufacture of SPION particles is magnetite, one of the most medically inert iron-based materials. According to the multilayer concept development process, the ability of iron oxide nanoparticles to be seen in the body using magnetic resonance imaging (MRI) is by far the most advantageous property of them for biomedical applications. The fine-tuning of the SPION's magnetic properties forms the foundation of the multilayer concept and can vary the surrounding water protons' rate of relaxation, changing the contrast. This feature has been successfully used in the detection and treatment of cancer 66,67. While, SPIONs engineered for application as nanotheranostics has been studied extensively for cancer treatment, some has been used for other diseases also. In this section we discuss application of some of the MRI-traceable nanotheranostics in different diseases.

#### SPIONs as nanotheranostics in cancer

**Figure 4:**
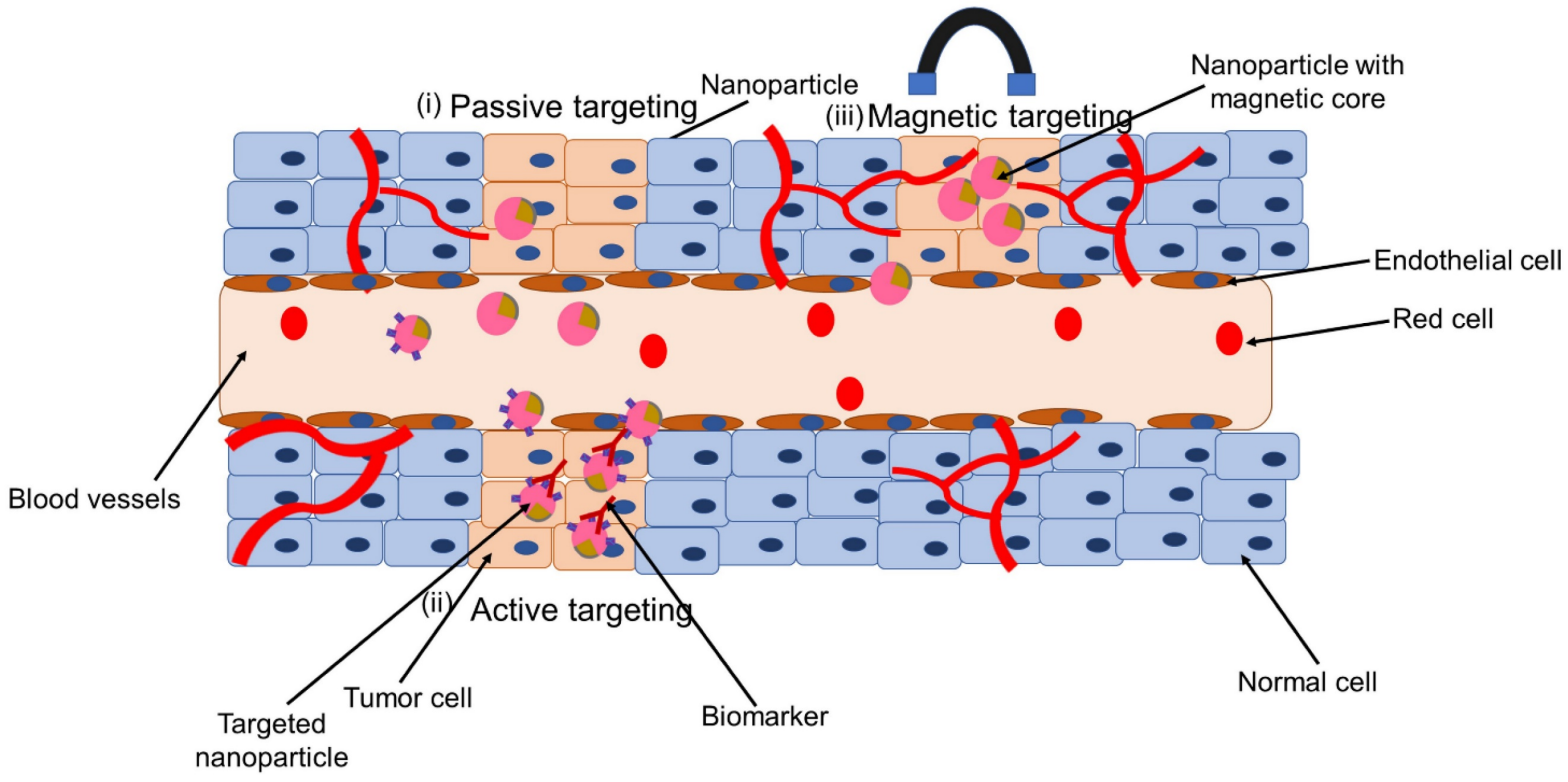
Application of SPION as nanotheranostics: (i) passive targeting, (ii) active targeting, and (iii) magnetic targeting

SPIONs targeted for nanotheranostics and cancer treatment can be engineered for passive targeting, active targeting, through externally applied magnetic field or dual targeting (Figure 4). In passive targeting, SPIONs selectively accumulate at the tumor site via enhanced permeability and retention (EPR) effect. Active targeting, on the other hand, utilizes specific interactions between a ligand on the surface of the nanoparticle and a biomarker, or receptor, on the target cell. The use of an external force, for example, magnetic targeting is applied in case magnetically responsive carriers are used. These are useful in case the diseased biological environment is very complex. The fourth type, dual targeting involves active and magnetic-targeting strategies, which has been explored to improve MRI detection and enhance anti-cancer efficacy compared to active or magnetic targeting alone. This fourth type has been tested in cancer or tumor treatment mostly 68. Details on the construction of nanotheranostics and their application in cancer is available in these reviews 44,69-71. In this section we discuss some few of the application of nanotheranostics in different types of cancer.

#### Nanotheranostics containing combination of chemotherapy and SPION in cancer

Chemotherapy is a standard treatment for a wide range of cancers. Chemotherapeutic drugs, however, have low tumor selectivity, dose-dependent toxicity, and poor water solubility. Another concern with chemotherapy is multidrug resistance (MDR), which is mostly attributable to the growth of the efflux pumps that remove cancer drugs from cell membranes. To overcome these constraints, nanodelivery systems that directly target cancer cells have been synthesized 72. Through passive targeting, nanoparticles gather in tumors as well as at inflammatory site through the EPR effect (Figure 4 (i)) 68. However, the EPR effect is now been widely acknowledged to be a highly heterogeneous phenomenon, particularly in cancer. There is high degree of variability regarding different tumor types between different patients and different tumors and metastases in the same patient. In clinical trials where chemotherapy-loaded nanomedicine formulations have been tested on patients, this variability is thought to be one of the main contributors to the diverse results. Iron oxide-based companion diagnostics can be used to visualize and quantify EPR effect in individual patients, and hence anticipate therapy responses. This is based on the co-localization of therapeutic and imaging nanoparticles (such as SPIONs) 68. This tailored technique might make it simple and affordable to distinguish between nanodrug therapy responders and non-responders. In this context, the intratumoral distribution of the fluorescently labeled nanoparticles PLGA-PEG and ferumoxytol was investigated through intravenous administration in nude mice implanted with HT1080 human fibrosarcoma xenografts. It showed co-localization of both particles. The same tumor model was used to investigate the accumulation rate of ferumoxytol with MRI. Based on that, it was able to predict the treatment efficacy of paclitaxel-loaded PGLA-PEG nanoparticles. This allowed it to anticipate the effectiveness of paclitaxel-loaded PGLA-PEG nanoparticles as a treatment. This concept was tested in the clinic. MRI was used to assess ferumoxytol accumulation in patients with metastatic solid tumors, and this was linked to the accumulation of iron oxide nanoparticles to the therapeutic response brought on by irinotecan-loaded liposomes (Onivyde®). According to the findings, ferumoxytol uptake may be a good and clinically relevant imaging biomarker for tracking the EPR effect and forecasting treatment outcomes for nanotherapy 16.

#### Nanotheranostics containing iron-oxide nanoparticles and cancer cell biomarkers

Numerous biomarkers of cancer have been considered as SPION targets. Active targeting utilizes specific interactions between a ligand on the nanoparticle surface and a biomarker or receptor on the target cell (Figure 4 (ii)) 68. For instance, the folate receptor, which is lacking in most normal tissues, is overexpressed in many different tumor types, including ovarian, breast, colorectal, renal cell carcinomas, brain metastases, and neuroendocrine malignancies. MRI results revealed that the tumor's average intensity decreased by 38% when folates were grafted onto SPIONs and delivered to the tumor site 69,73.

The transferrin receptor (TfR) is an additional target. Numerous studies have demonstrated that, as compared to normal cells, cancer cells frequently contain higher amounts of TfR. Breast cancer, bladder transitional cell carcinomas, prostate cancer, gliomas, and chronic lymphocytic leukemia have all been linked to increased TfR expression. T2-weighted images showed a 40% shift in signal intensity after injecting tumor bearing-mice with human transferrin proteins attached to SPIONs 34.

In a different experiment, SPIONs were coated with folate. Folate-SPIONs were taken up by HeLa cells, which express the folate receptor, and they significantly lowered signal intensity, creating a negative contrast. The entire result depended on the dose 31.

Prostate cancer cell lines express prostate specific membrane antigen (PSMA). Anti-PSMA Ab-SPIONs were used to treat LNCaP cell lines (J591 mAb-conjugated SPIONs). The imaging intensity in this cell line sample was reduced by 95% as a result of the treatment, which was detected using MRI imaging. These results suggested that target specific SPIONs can be used to improve treatment and prevent extraneous interactions in prostate cancer 31,42.

Making SPION into a photo thermal therapy (PTT) substance is another effective technique to use them as MRI agents and targeted treatment. In a breast cancer model, HA (Hyaluronic Acid)-SPIONs coupled with anti-CD44 (Cluster of Differentiation 44) antibodies have been studied. After the injection, a T2-weighted MRI image in vivo had around 40% more contrast than the initial state 1.5 hours later, and the effect persisted for 24 hours. Additionally, the PTT therapy with these systems enabled the tumor volume to be decreased from almost 4000 mm3 to a nearly unnoticeable level. These observations indicated the effectiveness of SPIONs' Ab-antigen-dependent endocytosis in cancer treatment 31,42.

Another intriguing instance of combining several components exists. The MUC-1 protein is encoded by the gene MUC1, which is located on the long arm of chromosome 1 at position 21. This region is frequently altered in pancreas, breast, lung, and colon cancer cells. The protein inhibits apoptosis process. Apoptosis causes cell death. But MUC1 alters and regulates different signaling mechanism to inhibit the apoptosis process and lead to tumor development, tumor progression and drug resistance 74. Multimodal imaging containing SPION, and other metals were constructed to treat MUC-1 expressing colon cancer cell line (HT-29). SPIONs were coated in gold in addition to being conjugated with MUC-1-specific aptamers. The primary tenet of that strategy was that since gold is a noble metal and is largely inert, it should stabilize SPIONs and lessen their cytotoxicity against healthy cells. In fact, aptamer-Au-SPIONs were substantially more poorly absorbed by the control cell line CHO (MUC1 negative) than by the HT-29 cell line. MR imaging revealed that SPIONs produced significant contrast enhancement in vitro. A further benefit of the gold coating was that aptamer-Au-SPIONs produced heat after being excited by an LED (Light-Emitting Diode), enabling photothermal therapy (PTT). This lead to a higher cell death of cancer cells compared to control cells 42,75.

Herceptin is a cancer-specific antibody that binds to the receptor tyrosine-protein kinase erbB-2 protein HER2/neu receptor present in breast cancer cells. In breast cancer cell line, SK-BR-3, overexpression of HER2/neu marker was observed. By using the 9-nm nanocrystals as model probe and functionalized with the conjugated ligand for Herceptin, images were obtained by T2-weighted MRI, which showed a significantly darker image with respect to the control cell line 42.

#### Theranostics iron oxide-based nanoformulations for cancer

The shell of iron oxide nanoparticles can for instance be modified with pharmacologically active compounds. This can either be done via chemical conjugation and a cleavable linker, or via physical intermolecular interactions. SPION can be loaded with many different therapeutic drugs. Especially anticancer agents have been employed, ranging from standard low molecular weight chemotherapeutics, such as doxorubicin, to more advanced therapeutic compounds, such as siRNA. In such setups, iron oxide nanoparticle-association prevents fast excretion or degradation, and it thereby increases the circulation time and the target site accumulation of the drugs. A multimodal-image guided siRNA delivery system was used where SPION was conjugated with siRNA, with additional modification of a membrane translocation peptide and with a near-infrared dye. With MRI and near-infrared in vivo optical imaging, accumulation of the theranostic material was evaluated in tumor, followed by gene silencing effect in 9L or LS174T (colon cancer cell line) tumor-bearing mice. Significant GFP and survivin silencing was seen in tumors. This research revealed innovative proof-of-concept for an SPION-based theranostic nanoplatform for siRNA delivery 16. In addition to colon cancer, siRNA based gene therapy was investigated for pancreatic cancer, liver cancer and lung cancer 68.

Table 3 shows example of some of the MRI-IONPs application in cancer.

#### Theranostic iron oxide nanoparticle-loaded microbubbles for cancer and neurological diseases

One of the obstacles in treating Alzheimer's disease, Parkinson's disease, and brain malignancies is effective and safe drug delivery across the blood brain barrier (BBB). The main obstacle to treat diseases of the central nervous system (CNS) is the blood-brain barrier (BBB). It is made up of tightly packed astrocytes, pericytes, endothelial cells, tight junctions, and extracellular matrix elements, which together line cerebral blood arteries and inhibit most hazardous and therapeutic substances from entering the CNS. These pathologies require systemic treatment with relatively large drug molecules such as growth factors and antibodies, which, due to an intact BBB, do not efficiently reach the target site upon I.V. injection. As a result, diseases of CNS such as Alzheimer's disease, Parkinson's disease, and brain tumors need to be considered for individualized and improved interventions. Therefore, it appears that the treatment of CNS diseases may benefit greatly from the development of materials and techniques that enable safe and effective drug transport across the BBB, ideally in an image-guided, targeted, and triggered manner. Microbubbles (MB) are microscopic vesicles that are filled with gas or air and have a lipidic, polymeric, or protein outer shell. MB are widely applied in a variety of biological applications. They are frequently utilized as intravascular contrast agents for contrast-enhanced ultrasonography (US) imaging, in addition to their direct and indirect uses in drug administration. Through a process known as sonoporation, the employment of US and MB in indirect drug delivery promotes the permeability of vascular endothelium and cellular membranes, facilitating the uptake of therapeutic chemicals at target sites and in target cells. In order to distribute drugs via image guidance, iron oxide nanoparticles can be loaded into the MB shell. Through the monitoring of BBB permeability, theranostic MB with iron oxide nanoparticles embedded in their shell have been used to enable effective and secure drug delivery across the BBB. In this approach, USPIO-loaded MB is destroyed upon exposure to US pulses, aiding BBB opening. The iron oxide nanoparticles are released from the MB shell during this process, extravasate across the permeabilized BBB, accumulate in extravascular brain tissue, and provide MRI information on the kinetics and degree of BBB opening. Dextran accumulation in the brain was found to correlate well with iron oxide nanoparticle-based MR imaging data, demonstrating the potential of such theranostic MB for image-guided drug delivery to the brain. Utilizing imaging guidance to reduce toxicity is particularly crucial in this situation 16.

Other than neurological diseases, for multimodality, image-guided cancer theranostics in single nanoplatforms, magnetic liposomes (MLs) were employed as in situ microbubble bombers 76. Liu et al. (2017) constructed a magnetic nanoliposome (AML) delivery system that contained hydrophobic anethole dithiolethiones (ADT), hydrogen sulfide (H_2_S) pro-drug, doped in the phospholipid bilayer, and superparamagnetic nanoparticles inside. After 6 hours of co-incubation with AMLs, HepG2 cells could be effectively bombed. The nanoscale AMLs could be intratumorally transformed to microsized H_2_S bubbles for in vivo applications after preferentially targeting the tumor tissue when spatiotemporally directed by an external magnetic field. Dual-modal imaging using magnetic resonance (MR) and ultrasound can be used to track this dynamic process. In addition, numerous microsized H_2_S bubbles can be continually produced by an enzymatic trigger at the same time as ADT molecules (organic H2S donors) are released in the tumor matrix. The localization of AMLs within the tumor microenvironment is made possible by such a nano-to-microsize shift, which also prevents their washout. The dynamic H2S microbubble generation process can also be determined using real-time ultrasound (US) imaging. Furthermore, using a higher ultrasound intensity for bubble cavitation, the H_2_S microbubbles acting as an intratumoral bomber might explode to obliterate the local tumor tissue while being directed by microbubble-enhanced US imaging. The anticancer effect may be enhanced by a synergistic interaction between the burst of microbubbles and the diffusion of intratumoral high-concentration H_2_S molecules in the deep tumor region. Due to the distinct ability of in situ microbubble creation, AMLs can significantly improve contrast in dual modalities of MR and US imaging. Additionally, when exposed to higher acoustic intensity, the intratumorally generated high-concentration H_2_S molecules can diffuse into the inner tumor regions to further have a synergistic antitumor effect. These molecules are imaged by real-time ultrasound imaging, which can first bomb to ablate the tumor tissue. AMLs treated with magnetic fields have shown extremely noticeably higher tumor growth inhibitions after a 7-day follow-up observation. Because of this, the intratumoral conversion of intricately designed nanostructures to microstructures has demonstrated enhanced anticancer activity, which may be encouraging for multimodal, accurate image-guided cancer therapy 76.

#### Theranostic iron oxide nanoparticle-containing materials in regenerative medicine

Iron oxide nanoparticles can be employed in regenerative medicine in a variety of ways, such instance as a cell-tracking tracer. Utilizing cells therapeutically opens new avenues for the treatment of diseases like cancer, neurological diseases, and immunological pathologies. In this regard, immune cells including T cells and dendritic cells as well as stem cells have been labelled with SPION to enable noninvasive tracking via MR imaging and to monitor in vivo transport at the site of action. By treating these cells with SPION in the presence or absence of a transfection agent, as well as progenitor cells, monocytes, and macrophages, labelling of these cells can be easily accomplished in vitro. Transfection might be required to encourage SPION uptake, depending on the phagocytic activity of the cell type under investigation. This is not necessary for phagocytes like macrophages, but for non-phagocytic cells like stem cells, cationic compounds like poly L-lysine or protamine are frequently used to boost the SPION absorption that is otherwise very low 16.

In cancer immunotherapy, dendritic cells are investigated for the activation of immune cells to induce the eradication of malignant cells. In these approaches, tumor antigens are loaded into dendritic cell vaccines, which, after successful injection into lymph nodes, will display the antigens to T cells. To assure the successful injection and distribution, autologous dendritic cells were labeled with SPION and ^111^ln-oxide and injected into the lymph node of melanoma patients. The fate of the injected cells was monitored using MRI and scintigraphy. In comparison to scintigraphy, MRI could provide a more precise visualization and confirmation of the dendritic cell vaccine administration. While the sensitivity of both techniques was comparable, only MRI was able to detect incorrect delivery (adjacent to rather than inside the lymph node) 16.

Numerous other diseases, such as neurological, musculoskeletal, and cardiovascular disorders, are being studied in relation to the use of stem cells for treatment. Monitoring the in vivo destiny of implanted or injected stem cells is crucial for enhancing knowledge and ensuring effective treatment. Brain ischemia, Parkinson's disease, Alzheimer's disease, and Huntington's disease are example of some of the diseases that may benefit from the use of neural progenitors and stem cells treatment. SPION-labeled stem cells have been investigated for treatment of some of the diseases. SPION-loaded labelled neural stem cells for traumatic brain injury and labeled pancreatic islet cells for diabetes have both been investigated in clinics. However, none of these theranostic ideas for regenerative medicine have yet been used in actual clinical settings 16.

Table 4 shows summary on the use of MRI-IONPs for different types of diseases.

## Challenges

### Toxicity

Despite having favorable outcomes for individuals, contrast agents like intravenous iron oxide are not currently being marketed. Even though ferumoxtran has a history of being safe and effective in diagnostics, its production has been halted. Ferumoxides and ferucarbotran, which had been previously approved for MRI were also withdrawn from the market. To address the clinical need, intravenous iron replacement agent ferumoxytol (Feraheme/Rienso, 17-30 nm particles coated with a low-molecular-weight semisynthetic carboxylated polymer) was used as a contrast agent to characterize the myocardial infarct pathology and differentiate between simple steatosis and nonalcoholic steatohepatitis (NASH). However, the concentration of ferumoxytol used for this purpose was very high for patients with myocardial infarct and liver disease. The high concentration possessed serious risk of anaphylactic response upon intravenous administration. As a result, FDA issued strongest type of warning for this product 77.

Another potential toxicity of SPIONs is that the small size (less than 5 nm) generates reactive oxygen species (ROS). Iron ions catalyze Haber-Weiss and Fenton reactions, which generate ROS. ROS could induce cell-damage of the healthy cells. NPs undergo two main processes that are important determinants of their cellular uptake. These are transient protein binding in culture media in vitro or body fluids in vivo and protein interaction the cell surface or membrane molecules. The coating of iron oxide core in SPION can affect its cellular uptake or total clearance from body. Moreover, MRI images from SPIONs in blood and those that were phagocytized by liver macrophages exhibited different patterns 77.

### In vivo validation

In vivo protein adsorption of NPs are a potential destabilizing contributor. Nanoparticles with polymeric surfactants are unstable in biological settings containing salts and proteins. Therefore, in vivo evaluations of IONPs are frequently difficult 43.

Another challenge for in vivo validation is the model to investigate the SPIONs effectiveness as contrast agent and nanotheranostics. For example, actual situation faced by cancer patients is substantially more diverse than that of animal models. None of the constructed and evaluated actively targeted and magnetic SPION have yet been used in the clinic, despite a wide variety of remarkable preclinical discoveries. Translational nanomedicine research must use patient classification to raise the possibility that therapy will be effective. Scientists and companies have realized this as the value of customized therapy and theranostics increases 42.

### Validation of imaging biomarkers

MRI has the advantage over other imaging modalities as it can be used to image the anatomical, structural, and molecular properties of the organ. In classic MRI, *T*_1_ and *T*_2_ relaxation times are used to monitor changes in tissues and these are unspecific. The magnitude of the effects may be variable and depend on organ and disease status. However, nanotheranostics relies on targeted delivery of the drug and imaging of the target molecule in addition to contrasting using MRI. Identification of MRI-based quantitative imaging biomarker (QIBs) can be useful to subtype patients as responders, non-responders, or patients with adverse drug toxicity. This can lead to patient tailored diagnostics and treatment, which is required for personalized medicine. However, there is no gold standard reference methods that could allow a purely experimental validation of an imaging biomarker. This is due to the complex relationship between biological properties and those that can be measured with MRI which need to cross multiple scales involving physical phenomenon in diverse physiological, biological, biomechanical, fluid dynamics and electromagnetism 20.

### Regulatory challenges

Acceptance of nanotheranostics and QIBs by clinics or regulatory bodies is another challenging issue. The reason is that there is still considerable knowledge gap on how a QIB will respond to treatment, alter with disease severity, and vary among patients. Moreover, it could be challenging to legitimize the large expenditure necessary for clinical validation in the absence of convincing theories 20.

### Economic challenge

The cost of the synthesis of nanotheranostics and cost of the MRI platforms are comparatively high, which can affect their market size and limit their use in limited-resource countries. Apart from this, development and formulation of patient specific treatment modalities are costly for biotech companies as these require several years for preclinical and clinical validation. Thus, there is increased financial risks with potential failure in clinical translation of NP formulation 78.

### Barriers in clinical translation

An obstacle to clinical translation is the fact that NP platforms are evaluated for effectiveness in large populations. Due to the enormous biological diversity of patients, the success of treatment might remain obscure for the actual subgroup of patients in which the treatment is successful. Stratified populations are more likely to respond uniformly to newly developed NP. Unfortunately, clinical trials are conducted among unstratified patients. To be successful, precision applications and more stratified trials are necessary 5.

### Biobanks in imaging and personalized medicine

Biobanks are considered as important instrument for personalized medicine. These were initially used for tissue or sample storage 18. However, later, context based biobanking have been evolved. Biobanks are mostly cohort based and are classified by clinical features such as, pathology, disease stage, therapy, and even healthy volunteer donated biobanks. Data available from biobanks are not only limited to the biospecimen collected now, but also can be used in future purpose with updated information. Samples collected and stored for biobanking need to follow standard operating procedures set by the International Society for Biological and Environmental Repositories (ISBER), which was founded in 2000. In addition to sample storage and processing, biobanking also keep records of an individual's genetics, proteomics, and metabolomics data. The purpose is to validate different information of a patient to identify disease related biomarkers. In this way, biobanks effectively support health care allowing the discovery and validation of disease biomarkers. Imaging biobanks are novel field of biobanks. Imaging biobanks can keep record of the patient's bioimages from advanced CT, MR and PET. Diagnostics images generated with cutting-edge imaging technologies can be used by high throughput computing to extract structural, functional, and molecular information in non-invasive manner and correlate to disease stage. Thus image data are useful for the identification and validation of non-invasive biomarkers, also known as imaging biomarkers (IB) and correlate imaging data with genetic data at the clinical setting 18. Despite the usefulness of imaging biomarkers that could improve a tailored treatment and diagnosis, no imaging biobanks are yest available.

## Conclusion

**Figure 5:**
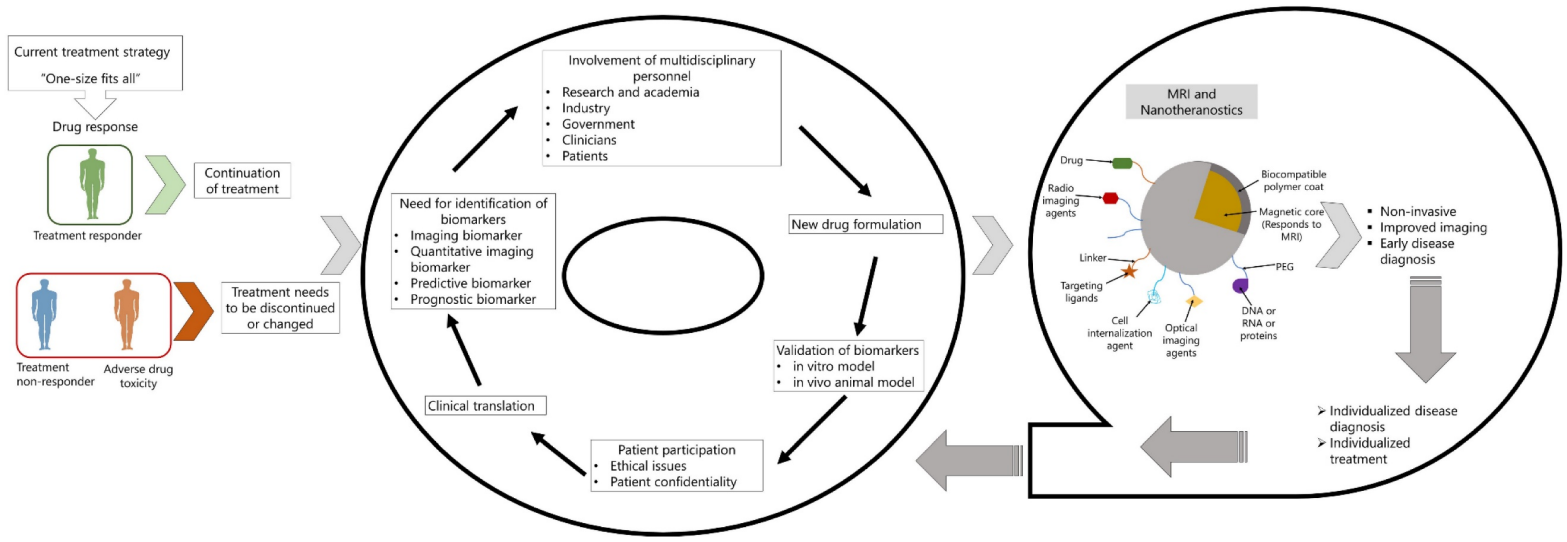
Factors associated with MRI-nanotheranostics for application in Personalized Medicine

The use of diagnostic imaging has increased dramatically in recent decades, with advanced cross-sectional imaging systems such as CT, MRI, as well as PET. However, imaging technology is challenging, and the cost of different imaging modalities is very high as the availability of imaging services around the world varies widely. Despite these challenges, imaging techniques used as screening tools for disease detection at subclinical levels enable coordinated preventative measures. Nanotheranostics, which combines nanotechnology and MRI, is developed so that therapy and diagnostics can be closely linked. Theranostic nanoparticles provide a useful way to learn about target site accumulation and biodistribution, which helps with patient pre-selection and the prediction of therapeutic results. While many research has been conducted in different areas of diseases applying MRI-IONPs basednanotheranostics, it is still in its early stages to be applied in personalized medicine. The success of PM depends on the identification of robust biomarkers that have predictive and prognostic value. MRI has advantage over other imaging modalities in its ability to image structural and molecular status. Over the past years, many MRI biomarkers have been reported. Detection of these imaging biomarkers using nanoparticles, especially, SPIONs require consideration of formulation of the nanoparticles and the attachment of the specific biomarker. Determining the correct response is important in personalized medicine (PM). In PM, it is important to determine whether treatment should be continued, adjusted, or terminated. In situations where treatment is ineffective, discontinuing or terminating treatment may provide patients with alternative therapies or spare them the side effects of ineffective therapies (Figure 5). Although the reliability and cost-effectiveness of screening are controversial, a personalized health care system is still needed. In this review, we have discussed application of iron-oxide nanoparticles as contrast agents during MRI imaging and in combination with drug delivery and imaging in one system. Despite the many different challenges, nanoparticles have huge potential to be used in personalized diagnosis, treatment and adequate drug delivery in complex diseases that affect heterogenous cell population or organ systems.

## Figures and Tables

**Table 1:** Properties of some of the iron oxide nanoparticles used in clinics

Name of IONPs	Coating	Size (nm)	Type of contrast	Applications	Route of administration	Reference
Ferumoxide	Dextran	120-180	Negative	Liver and spleen imaging, CNS imaging, cell labelling	Injectable solution	(16,35)
Ferumoxytol	Carboxymethyl dextran	3	Positive	Angiography; CNS imaging, macrophage imaging, cellular labelling, lymph node imaging	Intravenous (I.V.)	
Ferumoxsil	Silica	300	Negative	Gastrointestinal imaging	Oral	
Ferumoxtran	Dextran	30	Positive/negative	Lymph node imaging, CNS imaging, macrophage imaging, cell labeling	Injectable solution	
Ferucarbotran SHU-555A	Carboxydextran	60-80	Negative	Liver/spleen imaging	I.V.	
Ferucarbotran SHU-555C	Carboxydextran	20-50	Positive	Perfusion lymph node bone marrow	I.V.	

**Table 2:** MRI-IONP based biomarkers used for cancer in clinics

Biomarker	Application	Characteristics	Contrast agent type	Reference
Detection and characterization	• BI-RADS • PI-RADS • LI-RADS	Lesion morphology	T2, T1	(19)
Staging	TNM staging	Tumor morphology, presence and number of nodes, metastases	T2, T1	
Response	Treatment response in solid tumor	Change in tumor size	T2, T1	

Abbreviations: BI-RADS is breast imaging reporting and data system; PI-RADS is prostate imaging and data system; LI-RADS is liver imaging reporting and data system

**Table 3:** MRI-IONPs applied for clinical trials, diagnosis and nanotheranostics in cancer

IONPs in clinical trial				
*Magnetic nanoparticle system*	*Application*	*Cancer type*	*Status of clinical trial*	*Reference*
SPION	MRI	Pancreatic cancer	Completed	(44)
Paclitaxel albumin-stabilized nanoparticle	Chemotherapy	Breast cancer	Completed	
IONP for magnetic hyperthermia	Magnetic hyperthermia and MRI	Prostate cancer	Completed	
USPIO nanoparticle-ferumoxytol	MRI	Head and neck cancer	Active	
**Iron-oxide nanoparticles engineered for dual imaging and therapeutic applications**				
***Magnetic nanoparticle systems***	***Detection method***	***Therapeutic applications***	***Cancer type***	***References***
Gold nanorod-capped magnetite core/mesoporous silica shell nanoparticles	MRI	Doxorubicin chemotherapy; PTT		(44)
Gold shell-core IONP	MRI; PAI	PTT	Breast	
Multicore IONP with CuS shell	MRI	MHT; PTT; PDT	-	
cRGD-functionalized Doxorubicin-conjugated and ^64^Cu labeled SPION	PET; MRI	Doxorubicin chemotherapy	Glioblastoma	
Indium-^111^ labeled Trastuzumab-Doxorubicin Conjugated, and APTES-PEG coated SPION	SPECT; MRI	Tumor suppression. Antibody and chemotherapeutic agents	Breast	
Manganese-doped iron oxide nanoparticles, coated with bovine serum albumin and functionalized with a cyclic Arg-Gly-Asp (cRGD) peptide and cy5 dye-labeled siRNA	MRI	Inhibition of Green fluorescence protein by the siRNA moiety, and interference of receptor-mediated endocytosis via targeting tumor cells overexpressed α_v_β_3_ integrin by RGD peptide	Breast	
Paclitaxel loaded, PEG modified liposome iron oxide MNP	MRI	Paclitaxel	Breast	
Liposome, ADT loaded iron oxide MNP, encapsulated with PEG	MRI	H_2_S	Liver	
Rituximab loaded liposome, iron oxide MNP, encapsulated with PEG	MRI	Rituximab	Brain Lymphoma	
**MRI traceable nanotheranostics with gene therapy**				
***Magnetic system***	***Targeting strategy***	***Gene***	***Cancer type***	***Reference***
SPIONs (T2 MRI)	EPPT1 peptide targeting uMUC1 and MPAPs	siPLK1	Pancreatic cancer	(68)
SPIONs (T2 MRI)	MT	HSV-TK-GCV	Liver cancer	
SPIONs (T2 MRI)	HA targeting CD44	Magnetized AAV2	Lung cancer	
SPIONs (T2 MRI)	RGD-modified PEG-grafted PEI functionalized with SPIONs	RGD	in vitro: human hepatocellular carcinoma cell line Bel-7402: in vivo: nude mice Bel-7402 hepatoma model	(31)
SPIONs (T2 MRI)	SPIONs coated with PEG-grafted chitosan and PEI	Chlorotoxin	in vitro: C6 rat glioma cells	
SPIONs (T2 MRI)	Iron oxide NP core coated with a copolymer of chitosan, PEG, and PEI. GFP encoding DNA was bound to these NPs, and chlorotoxin was then attached using a short PEG linker	Chlorotoxin	in vivo intravenous injection to mice bearing C6 xenograft of glioma tumor	
SPIONs (T2 MRI)	Magnetic NP-EPPT-siRNA	EPPT peptide that specifically targets uMUC-1	in vivo intravenous delivery into subcutaneous mouse models of breast cancer	
SPIONs (T2 MRI)	VP4-coated Fe_3_O_4_ NPs loaded with doxorubicin	Rotavirus capsid surface protein VP4	MR and fluorescence imaging and drug delivery of human hepatic carcinoma	
SPIONs (T2 MRI)	scAbCD3-PEG-grafted PEI complexed with SPIONs further condensed with plasmid DNA	scAbCD3	in vitro: HB8521 cells, a rat T-lymphocyte line	
**MRI traceable nanotheranostics with combination therapy**				
***Magnetic system***	***Target molecule***	***Cancer type***	***Reference***	
Iron ion T2 MRI with ICG, PTT	CD44	Breast cancer cell line	(68)	
SPION T2 MRI, Chemo-PTT	EGFR	Lung cancer cell line		
SPION T2 MRI, Chemo-US	FA	Liver cancer cell line		
SPION T2 MRI, Chemo-MHT-PTT	MHI-148	Colorectal cancer cell line		
SPION T2 MRI with Cy5.5 or FITC (FI), Chemo gene therapy	IL-4R	Breast cancer cell line		
IONP T2 MRI with cyanine, PTT	TR	Lung cancer cell line		
MRI and drug delivery	Folate	in vitro, folate receptor overexpressing HeLa and normal L929 fibroblast cells, cervical cancer	(31)	
MRI and drug delivery	Folate	in vitro, HeLa cell line, cervical cancer		
MRI and drug delivery	Folate	in vitro, HeLa cells, cervical cancer		
MRI and drug delivery	Folate	in vivo, rats and rabbits, liver cancer		
MRI and drug delivery	cRGD peptide	in vitro, U87MG cells, human glioblastoma		
MRI and drug delivery	Chlorotoxin	in vitro, C6 rat glioma cells		
MRI-guided drug delivery	CG-rich duplex containing PSMA aptamer	in vitro and in vivo, LNCaP cells, prostate cancer		
Drug delivery and MRI	A10 RNA aptamer which binds the extracellular domain of the PSMA	in vitro, LNCaP and PC3 cells, prostate cancer		
MR and fluorescence imaging	Antibody HuCC49 DCH2 and fluorescent dye 5-FAM	in vitro, LS174T colon cancer cell line		
Drug delivery and MRI	Monoclonal antibody against the neu receptor	in vivo, transgenic MMC cells		
MRI scanning of F3 targeted nanoparticle	F3 targeted nanoparticle	in vivo, rats implanted with gliosarcoma (9L) cells into the right forebrain		

**Table 4:** Summary on the application of MRI-IONPs in different types of diseases

Disease type	Diagnostics features	Reference
***Cellular and structural visualization***		
Cancer	• Clinical imaging of liver and spleen tumor metastases through RES-mediated uptake of IONPs • Effective identification of lymph node metastases • Prolonged delineation of brain tumor boundaries and quantify tumor volumes • Imaging of CNS tumor neovasculature and assessing therapeutic response to antiangiogenic chemotherapeutic agents	(30)
Liver disease	Monitor Kupffer cell function using MR imaging in obstructive jaundice	(58)
Central nervous systems (CNS) disease imaging	• Diagnosis and staging of multiple sclerosis (MS) • MRI visualization of neuroinflammation in vivo • Monitoring of leukocyte (macrophage) trafficking in the brain	(16)
Autoimmune diseases	Monitor insulitis via imaging pancreatic inflammation for type 1 diabetes (T1D)	(16)
Cardiovascular disease	• SPION uptake by macrophages helps to visualize vascular inflammation in atherosclerotic plaques • Evaluation on the risk of ischemic stroke	(58,63)
Sepsis	Reactive oxygen species (ROS) tracking by imaging	(64)
Septic arthritis	Macrophage imaging helped to visualize knee infection	(58)
Osteomyelitis	Difference between infectious osteomyelitis and vertebral inflammation could be obtained	(58)
***Cell tracking and cell therapy***		
Cancer	Autologous dendritic cells used as cancer vaccine was loaded with tumor antigen and labeled with SPION and 111In-oxide. It helped to visualize lymph node and vaccine administration in melanoma patient	(16)
Neuroinflammation	SPION labeled neural stem cells helped in the treatment and visualization of traumatic brain injury.	(16)
Autoimmune disease	SPION labeled pancreatic islet cells helped to investigate diabetes	(16)
***Molecular imaging***		
Cancer	Allows sensitive and specific monitoring of key molecular targets Allows host's immune response at different stages of cancer	(68)
